# Specificity of Affective Responses in Misophonia Depends on Trigger Identification

**DOI:** 10.3389/fnins.2022.879583

**Published:** 2022-05-26

**Authors:** Marie-Anick Savard, Anastasia G. Sares, Emily B. J. Coffey, Mickael L. D. Deroche

**Affiliations:** ^1^Department of Psychology, Concordia University, Montreal, QC, Canada; ^2^Laboratory for Brain, Music and Sound Research (BRAMS), Montreal, QC, Canada; ^3^Centre for Research on Brain, Language, and Music (CRBLM), Montreal, QC, Canada

**Keywords:** misophonia, auditory cognition, emotion regulation, anxiety, anger, mental health, sound sensitivity

## Abstract

Individuals with misophonia, a disorder involving extreme sound sensitivity, report significant anger, disgust, and anxiety in response to select but usually common sounds. While estimates of prevalence within certain populations such as college students have approached 20%, it is currently unknown what percentage of people experience misophonic responses to such “trigger” sounds. Furthermore, there is little understanding of the fundamental processes involved. In this study, we aimed to characterize the distribution of misophonic symptoms in a general population, as well as clarify whether the aversive emotional responses to trigger sounds are partly caused by acoustic salience of the sound itself, or by recognition of the sound. Using multi-talker babble as masking noise to decrease participants' ability to identify sounds, we assessed how identification of common trigger sounds related to subjective emotional responses in 300 adults who participated in an online study. Participants were asked to listen to and identify neutral, unpleasant and trigger sounds embedded in different levels of the masking noise (signal-to-noise ratios: −30, −20, −10, 0, +10 dB), and then to evaluate their subjective judgment of the sounds (pleasantness) and emotional reactions to them (anxiety, anger, and disgust). Using participants' scores on a scale quantifying misophonia sensitivity, we selected the top and bottom 20% scorers from the distribution to form a Most-Misophonic subgroup (*N* = 66) and Least-Misophonic subgroup (*N* = 68). Both groups were better at identifying triggers than unpleasant sounds, which themselves were identified better than neutral sounds. Both groups also recognized the aversiveness of the unpleasant and trigger sounds, yet for the Most-Misophonic group, there was a greater increase in subjective ratings of negative emotions once the sounds became identifiable, especially for trigger sounds. These results highlight the heightened salience of trigger sounds, but furthermore suggest that learning and higher-order evaluation of sounds play an important role in misophonia.

## 1. Introduction

Misophonia is a disorder (Swedo et al., 2022) involving extreme sensitivity to common sounds such as eating, smacking lips, or breathing (Schröder et al., 2013; Jastreboff and Jastreboff, 2014) which trigger a strong negative emotional state. The misophonic response typically involves irritability, annoyance, anxiety, disgust, and anger when people are exposed to their trigger sounds (Rouw and Erfanian, 2018). People with misophonia show heightened trigger-specific physiological autonomic responses, experience a strong desire to escape from the environment where they hear trigger sounds (Kumar et al., 2014), and can sometimes feel a desire to harm those producing the sounds (Edelstein et al., 2013). As a consequence, they tend to avoid situations where triggers are likely to be encountered (e.g., social gatherings, classrooms, family dinners, etc.) (Schröder et al., 2013). These avoidance behaviors can be detrimental to wellbeing, education, and social relationships (Neal and Cavanna, 2013; Jager et al., 2020), which highlights the need to better characterize misophonia, and explore the underlying mechanisms by which sounds cause such strong reactions in certain people.

### 1.1. Misophonia in a General Population

Misophonia within general populations has only recently become a focus of scientific inquiry. Studies in large samples (*N* > 300) estimate that, when assessed with scales specifically designed to target misophonia, about 12–20% of people suffer from moderate or severe misophonia symptoms, cross-culturally (Turkey, United Kingdom, United States, China) (Wu et al., 2014; Zhou et al., 2017; Kılıç et al., 2021; Naylor et al., 2021). Moderate and severe symptoms tend to be grouped together, and are characterized by the interference that they cause in daily life at work, school, and home. Research on the prevalence of misophonia labels subjects as having misophonia or not based on cut-off points; however, it is unknown if those with severe symptoms of misophonia are truly a categorically special population or merely the tail end of a continuum of sound-sensitivity symptoms. Adding to this incertitude, there is still little knowledge of how prevalence may differ between biological sexes. Although some research suggests that misophonia is more prevalent in females, the samples on which these observations were based were primarily comprised of female university students (67–84% female), which the authors highlight as a limitation to the generalizability of their results (Wu et al., 2014; Zhou et al., 2017; Kılıç et al., 2021). As such, it is not yet clear if the apparent imbalance is caused by sample bias, noting that most studies recruit within psychology departments, or due to a real difference in prevalence across sexes. In addition to sex, age is a factor of interest in the study of misophonia, as findings tend to point toward younger age being associated with greater rates of misophonia. Indeed, researchers find a higher proportion of individuals with misophonia in younger samples (mean age 19.8 in Zhou et al., 2017 and 21.4 in Wu et al., 2014) than in relatively older and more age-balanced samples. In a study with participants who were more balanced in age (age range of 15–88 in Kılıç et al., 2021, mean age of 43.5 years old), researchers found lower average prevalence of misophonia and observed that younger age was related to higher rates of misophonia. Though prevalence estimates are influenced by the measurement tools and degree of severity considered as a cut-off, it is clear that there are a large number of sufferers globally. A better understanding of who suffers from misophonia is needed.

### 1.2. Misophonia, a Sound-Specific or Person-Specific Disorder?

To understand why certain sounds cause such strong reactions in people with misophonia, some researchers turned their attention to the nature of trigger sounds. Although they tend to vary between individuals, there are commonalities in the categories of sounds reported as triggers. Specifically, they are often everyday sounds created by other individuals (and occasionally animals), and sometimes repetitive environmental sounds (Schröder et al., 2013, 2017; Kumar et al., 2014). One study found that in a large misophonic sample (*N* = 575), most participants were triggered by eating sounds (96% of the sample), nasal and breathing sounds (85%), repetitive tapping (74%), and mouth/throat sounds (60%) (Jager et al., 2020). One observation about the nature of trigger sounds is that they tend to share some acoustic properties. Whether they are of organic (e.g., eating) or non-organic (e.g., clock ticking) origin, triggers still tend to be pattern-based and repetitive (Vitoratou et al., 2021). In general, sounds that are temporally modulated tend to stand out from a noisy background (Kayser et al., 2005); this seems particularly exacerbated in some modulation rates resulting in a sense of roughness (Arnal et al., 2019), while other work showed an association between ratings of unpleasantness and temporal modulation (i.e., 1–16 Hz) in naturalistic sounds (Kumar et al., 2008). Such acoustic qualities make auditory stimuli easier to detect, grab the listener's attention, and are thought to be processed *via* bottom-up auditory mechanisms (Duangudom and Anderson, 2007). Given that typical trigger sounds seem to share these attention-grabbing properties, it is possible that early-attentive processes are somehow involved in misophonia (an idea discussed in Schröder et al., 2014, discussed in Section 4.2). If it is true that misophonic triggers are easier to detect than other types of sounds, it may be that such bottom-up cues are involved in the development of these sounds as triggers. In other words, more acoustically salient stimuli would be more likely to become triggers, because people are better at detecting them.

At higher levels of processing, the meaning of stimuli is extracted as we recognize sounds, interpret them, and make links with previous memories. Another line of research thus investigates the common observation that individuals' trigger sounds seem to relate primarily to contextual cues, and only partially to physical characteristics of the sounds (Jastreboff and Jastreboff, 2001). Evidence from past studies points toward the involvement of higher-level evaluation of sounds in the misophonic response. Such features include the meaning, social context, and interpretation of the sound (Schröder et al., 2013; Bruxner, 2016), the belief that the sound is a potential threat or that exposure to it will be harmful (Jastreboff and Jastreboff, 2014), and the influence of personality traits (Daniels et al., 2020). In addition, a majority of people with misophonia report that their responses are exacerbated if the sounds are produced by certain people, often close friends, coworkers, and family members; or that their misophonic responses may even be isolated to these events (Edelstein et al., 2013). This insight about the importance of the person who produces the sound in the misophonic response supports the involvement of higher-level cognitive appraisals (i.e., subjective interpretation) in the misophonic reaction. Furthermore, individuals with misophonia report that they may react to a given sound in one setting (such as in their home) but not react as strongly to the same sound in another setting (such as in the home of a friend) (Jastreboff and Jastreboff, 2014). Considering that the sounds share similar acoustic properties regardless of who or what produces them, it is likely that a person's appraisal of a sound and context around it affect whether a misophonic reaction is produced or not. This involvement of top-down processes has been hinted at by self-reports and case studies, and briefly mentioned in Hansen et al. (2021), however it has yet to be supported by behavioral assessments.

With evidence for both bottom-up and top-down mechanisms being involved in misophonia, disentangling the factors contributing to the misophonic response and their relative importance in producing said response will lay essential groundwork for devising effective intervention strategies. If evidence supports a greater importance of acoustic cues, then solutions should turn toward modifying the acoustic properties of triggers. If evidence supports the importance of higher order evaluations of sounds, then solutions must focus on the associative link with the specific triggers of each patient and imagine ways in which one could break those associations.

### 1.3. Goal of the Present Study

The first aim of this study was to characterize the distribution of misophonia symptoms in a general population. To do so, we collected responses from an online community sample on a scale assessing misophonia. We then examined the shape of the distribution to determine whether people with severe misophonia symptoms represent a different group than those with sub-clinical symptoms (binomial distribution) or if they are simply the tail-end of a normal distribution of misophonia sensitivity. This first aim was descriptive in nature. In terms of a potential difference between males and females, we tested the hypothesis that scores on the measure designed to screen for misophonia would differ between sexes. Based on previous research (Wu et al., 2014; Zhou et al., 2017; Kılıç et al., 2021), we expected that females would score higher than males, thus reporting more misophonia symptoms.

The second aim of this study was to determine whether misophonia could be partly caused by a reaction to acoustic salience of typical trigger sounds regardless of what the sound is (bottom-up processing), or if a sound must be consciously identified as a known trigger in order to cause a misophonic response (top-down evaluation of sounds). To do this, we selected subgroups of most- and least-misophonic participants and measured identification thresholds (i.e., the point at which sounds from a category were identified by a given participant) of both groups for different categories of sounds (neutral sounds, unpleasant sounds, typical misophonic triggers). To establish if trigger sounds were indeed more acoustically salient (i.e., more attention-grabbing) than other categories of sounds, we asked participants to identify sounds in the presence of masking noise with different signal-to-noise ratios (SNR). We then explored the role of sound identification on subjective emotional responses (anger, anxiety, disgust) and perceived pleasantness of the sounds, by comparing sub-threshold and supra-threshold responses. This second aim yielded three different hypotheses. The first hypothesis, related to lower-level properties of trigger sounds, is that as SNR increases (and all sounds become easier to detect), there would be a difference in identification of the different sound categories, such that trigger sounds would be identified more easily than neutral and unpleasant sounds. The second hypothesis, related to individuals' differing ability to detect trigger sounds, was that the group of people most prone to misophonia would have lower detection thresholds for trigger sounds (i.e., they would be able to detect trigger sounds at a lower SNR level). A third hypothesis, related to the potential involvement of higher-order processes in subjective emotional responses of most- and least-misophonic individuals, was that the differences in subjective ratings of trigger sounds would only appear after the sounds are identified. In other words, at an SNR level where sounds are not identified, both least- and most-misophonic groups would have similar subjective ratings of sounds, and at an SNR level where the sounds are identified, responses would differ between groups for trigger sounds.

This study will further our understanding of who suffers from misophonia, whether common trigger sounds differ from other environmental sounds in their salience, and if this potential effect of acoustic properties or higher order evaluation of sounds play important roles in misophonic responses.

## 2. Methods

### 2.1. Participants

A total of 300 adults participated in this study (149 males, 151 females; age range: 18–50 years, mean age: 24.6 *SD* = 6.7) and were recruited online through Prolific (https://www.prolific.co/). Prolific is an online platform which combines decent recruitment standards with reasonable cost, and allows researchers to pre-screen participants based on information provided when the participants sign up to the platform, which is updated over time (Palan and Schitter, 2018). Research comparing Prolific to other more widely used platforms (e.g., MTurk) showed that Prolific provides the highest quality data; participants on Prolific generally devote more attention to the tasks, comprehend instructions better, answer questionnaire items more carefully, and behave more honestly than on comparable platforms (Eyal et al., 2021).

To ensure that participants had a level of English fluency that would allow them to understand and take part in the study, recruitment was only open to residents of predominantly English-speaking countries (65% from Canada/USA, 32% from the Ireland/UK, 3% from Australia/New-Zealand). The participants came from 48 different countries of origin, with general representation as follows: 52% from North America, 30% from Europe, 13% from Asia, 2% from Africa, 1% from South America, 1% from Oceania (1% missing data). From the resulting sample, 64% were English monolinguals, 28% were bilinguals, and 8% were fluent in three to five languages (including English). Furthermore, students and non-students were represented in our sample (students: 60%, non-students: 40%), in addition to people with differing employment status (full-time: 31%; part-time: 22%; unemployed: 23%; other: 24%). Subgroups were defined at the data analysis stage based on total MisoQuest scores (Siepsiak et al., 2020a). See Section 3.1 for details regarding the grouping approach.

All participants reported being in good neurological health with normal hearing and were free of any diagnosed language disorder. Given that comorbidity of misophonia with psychiatric symptoms could contribute to high levels of anxiety or anger, we used the Prolific pre-screeners to exclude all individuals who were taking medication to treat symptoms of depression, anxiety, or low mood. This was done to limit the number of individuals experiencing severe psychiatric symptoms in our sample. The exclusion criteria also included a diagnosis of Autism Spectrum Disorder (based on participant self-report on Prolific), as this disorder shows considerable overlap with misophonia in terms of symptomatology related to sound sensitivity (Stiegler and Davis, 2010). As in other crowdsourcing platforms used for behavioral experiments, Prolific provides an approval rate for each participant. All participants who completed our study had an approval rate above 92% (*M* = 99.3, *SD* = 1.8), which we considered to be an acceptable range. No other exclusion criteria were specified for this study. All participants gave informed consent and were compensated with 2.95GBP (equivalent to $5CAD) for their time through Prolific. The experimental protocol was approved by Concordia University's Human Research Ethics Committee.

### 2.2. Protocol

Participants were recruited through Prolific (www.prolific.co), and redirected to an online behavioral platform (Pavlovia, https://pavlovia.org/) running the experiment designed on PsychoPy (Peirce et al., 2019). The entire experiment took on average 28 min to complete.

Before proceeding with the task, participants were asked to complete a questionnaire designed to screen for misophonia (see Section 2.4). They were also asked to specify the type of audio output that they were using (earbuds, headphones, default computer audio, speakers), and rate the quality of their audio (from 1 = poor to 5 = excellent). Note that all participants rated their audio quality as a 3 or higher.

Participants were presented with written instructions on how to complete the task and completed three practice trials. The sounds in the practice trials were presented without masking noise. After each sound, participants rated their subjective responses to the sound presented and identified it as they would in the task. This allowed participants to familiarize themselves with the scales for subjective ratings and with the labels for the forced-choice identification task, which they were prompted to closely examine. For the first two practice trials, they heard sounds of a Toddler Crying (1st trial) and a Washing Machine (2nd trial) for 15 s with a prompt to take this time to adjust the audio to be comfortably loud. In the third practice trial, participants were informed that the sounds in the study would be considerably shorter (3 s rather than 15) and proceeded to a trial containing a 3-s version of an Eating sound (different from the Eating sound used in the task). Feedback questions at the end of the study revealed that all participants found the instructions clear and had no problem understanding the task.

**Figure 1:**
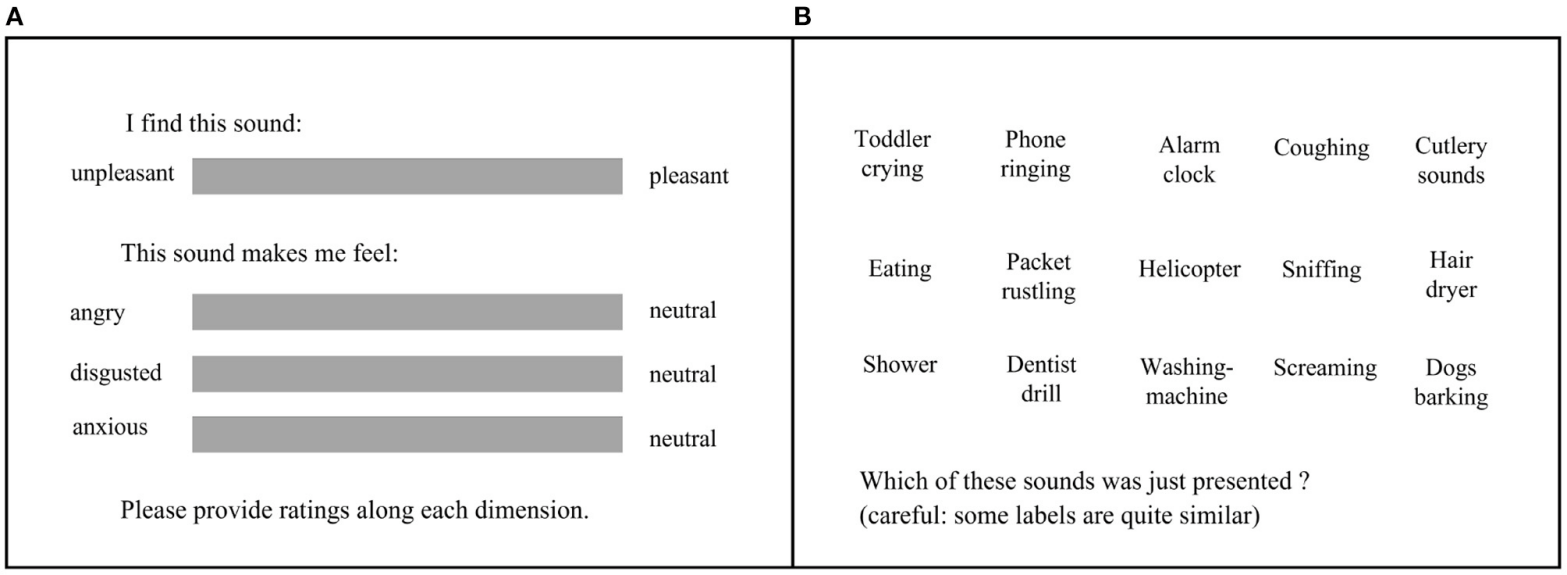
**(A)** Subjective ratings. Participants had unlimited time to rate the pleasantness of the sound (from unpleasant to pleasant) and the subjective feelings of anger, disgust, and anxiety after hearing the sound. **(B)** 15-alternative forced-choice task. Participants had unlimited time to click on the label corresponding to the sound that they just heard.

The task itself consisted of 75 trials, where neutral, unpleasant, and trigger sounds were embedded in multitalker babble (see Section 2.3), with different levels of signal-to-noise ratio (SNR; 15 sounds × 5 SNR levels). For each trial, participants first listened to the stimuli (3 s), then were prompted to rate the pleasantness of the sound (from 0 = unpleasant to 100 = pleasant), as well as their subjective anger (from 0 = angry to 100 = neutral), disgust (from 0 = disgusted to 100 = neutral), and anxiety (from 0 = anxious to 100 = neutral), using sliders on a continuous scale. In contrast with appraisal of the sound itself, the person-centered metrics were unidirectional with a neutral state on one end and the negative affect on the other, such that negative to neutral reactions were captured. Finally, for each trial, participants completed a 15-alternative forced-choice (15-AFC) task, where they were presented with labels describing all the possible sounds and were asked to identify the one that they had just heard. The experimental interface is presented in Figure 1. The participants completed all 75 trials in one single block, and the order of sounds was fully randomized within the block.

After the experimental portion ended, participants were also asked to describe what they thought the purpose of the experiment was and whether they had noticed anything particular about the sounds (open-ended response). Of the 300 participants, a total of 19 participants provided vague answers to both these questions. We visually assessed the data for these individuals to check that they had done the task correctly. Because they all showed good identification of the sounds (above 80% identification) on trials where the sounds were louder than the babble, we concluded that all participants were likely to have been engaged throughout the experiment. Participants were further asked whether they had experienced technical difficulties; no participant reported difficulties preventing them from completing the task.

### 2.3. Stimuli

The neutral, unpleasant, and misophonic trigger sounds used in this study were borrowed from Kumar et al. (2017). The materials originally comprised 42 sounds (each category consisting of 14 stimuli) of 15 s in length, from which we selected a subset of 15 sounds (5 from each category). The triggers in this set of sounds are related to orofacial actions (eating, drinking, etc.), which is in line with previous assessments of misophonia showing that orofacial sounds are the most common misophonic triggers. Indeed, Jager et al. (2020) found that all participants in their large (*N* = 575) sample had at least one oral or nasal sound as a trigger, and Vitoratou et al. (2021) showed that people with misophonia were more than 40 times more likely than those without misophonia to be triggered by eating sounds, and more than 20 times more likely to be triggered by loud/unusual breathing sounds. Though we understand that misophonia is characterized by a wider range of trigger sounds (as shown in Daniels et al., 2020), we used human-generated sounds related to orofacial actions in the present study, given that they are the most common triggers among individuals with misophonia.

Because the study design involved a total of 75 trials (5 SNR levels for each of the 15 sounds), we decided to select a representative 3 s clip from each, to reduce the length of the experiment and avoid participant fatigue. The fifteen sounds were selected based on pilot testing. Sounds were eliminated if they were frequently confused with another sound (e.g., Vacuum Cleaner and Hair Dryer), if they had highly similar semantic meaning (e.g., Eating, Chewing, and Crunching), or if they were difficult to identify in their 3-s form (e.g., Kettle Boiling, the acoustic properties of which evolved over 15 s). The final set of sounds comprised: Hair Dryer, Helicopter, Phone Ringing, Shower, and Washing Machine as neutral sounds; Alarm Clock, Dentist Drill, Female Scream, Multiple Dogs Barking, and Toddler Crying as unpleasant sounds; Coughing, Sniffing, Eating, Packet Rustling, and Cutlery as trigger sounds.

Multi-talker babble is often used as a masker for speech perception and hearing-in-noise experiments (Coffey et al., 2017). It is a type of noise that many listeners encounter on a regular basis in everyday life, therefore it has a higher degree of ecological validity than other types of maskers (Silbert et al., 2014). Because trigger sounds are frequently human-generated and heard in social contexts, we also adopted it as a masker.

In this study, we used 10-talked babble, with different levels of SNR. The levels were chosen via piloting such that, at the lowest level, the target sound would be generally indistinguishable among the babble, whereas at the highest level, the target sound would be very easily detectable from the babble. Thus, the chosen levels were covering most of the underlying psychometric function from which an inflection point could be well-bracketed. The resulting SNR levels were: −30, −20, −10, 0, +10 dB.

### 2.4. Questionnaires

The MisoQuest (Siepsiak et al., 2020a) is a 14-item questionnaire designed to screen for misophonia as a disorder in which a person is “triggered immediately by certain sounds, with anger as a core (but not exclusive) emotion”. The questionnaire includes items assessing different aspects of misophonia, from basic phenomenology (e.g., “I find some sounds made by the human body unbearable.”), to clinically-relevant questions about avoidance behavior and daily functioning (e.g., “If I can, I avoid meeting with certain people because of the sounds they make.”). For each item, participants were required to answer on a 5-point Likert-scale (from 1 = completely disagree, to 5 = completely agree). Misophonia symptomatology is indicated by summing the scores together, for a maximum of 70 points. The MisoQuest was developed in Polish with an Exploratory Factor Analysis (EFA), and showed excellent reliability (Cronbach's alpha = 0.955) in a misophonic sample. The English translation was provided by the team who developed the questionnaire, and (to the best of our knowledge) has yet to be validated in an English-speaking sample. The internal consistency (Cronbach's alpha) of the MisoQuest in our sample of 300 participants was of 0.890.

### 2.5. Statistical Approach

The first analysis concerned the prevalence of misophonia-like symptoms in our online community sample. We characterized the four moments of the distribution of MisoQuest scores (mean, standard deviation, skewness, and kurtosis), and used a Shapiro–Wilk test to examine the normality of this distribution. We reiterated this analysis split by sex (male or female), and regressed the MisoQuest scores by chronological age. A further exploration of sex effects on each item of this questionnaire was conducted with non-parametric *t*-tests (given the ordinal nature of the DV on an item basis) and corroborated by a Bayesian approach. This helped isolate which aspects of the questionnaire were likely to depend on sex, and which were not. Finally, the distribution of MisoQuest scores was divided in the top and bottom 20% to form two sub-groups: Most- and Least- Misophonic. Note that this was a critical step to the rest of the analyses, which focused on these two subgroups.

The second analysis concerned the performance in the identification of each sound with one of the 15 labels. A sigmoid function was fitted to the percent correct score, averaged for each category (neutral, unpleasant, and trigger sounds) across the five SNRs (from −30 to +10 dB). From these fits, a threshold was extracted at a fixed level of performance of 60.5% (which corresponds to d' of 2 in a 15-AFC task). This threshold was then submitted to a mixed analysis of variance (ANOVA) with one between-subject factor (most- vs. least-misophonic group) and one within-subject factor (category: neutral, unpleasant, trigger). Greenhouse-Geisser corrections were applied to effects and interactions that violated the assumption of sphericity. *Post-hoc* pairwise comparisons further explored the effect of category, correcting the inflation of type I error with Bonferroni adjustments.

The third analysis concerned the subjective ratings, collected for each of the five sounds in each of the three categories of sound, at each of the five SNRs (like the performance data). These ratings were fitted with a second-degree polynomial as a function of SNR. The position of the threshold divided the SNR scale in windows where sounds were or were not identifiable. The subjective ratings were averaged from the fits in each of these two windows, providing two values (sub-threshold rating and supra-threshold rating). The goal of this third analysis was to determine whether the supra-threshold rating would depart substantially from the sub-threshold rating, specifically for trigger sounds and specifically for the most-misophonic group. Thus, these ratings were submitted to a mixed ANOVA with one between-subject factor (group) and two within-subject factors (SNR window: sub- vs. supra-threshold, and category: 3 levels). To further explore the 3-way interaction, the change in rating (sub- vs. supra-threshold) was calculated and submitted to a 2-way ANOVA similar to that described above (second analysis). With this reduced design, the simple effect of group separately for neutral, unpleasant, and trigger sounds enabled us to point at the type of sound that could elicit a particularly aversive experience (induced by the sound becoming identifiable) in the Most-Misophonic group. Finally, note that this third approach was repeated in four different versions, for (1) unpleasantness, (2) anger, (3) disgust, and (4) anxiety, and were described as identically as possible.

## 3. Results

### 3.1. MisoQuest Scores

**Figure 2:**
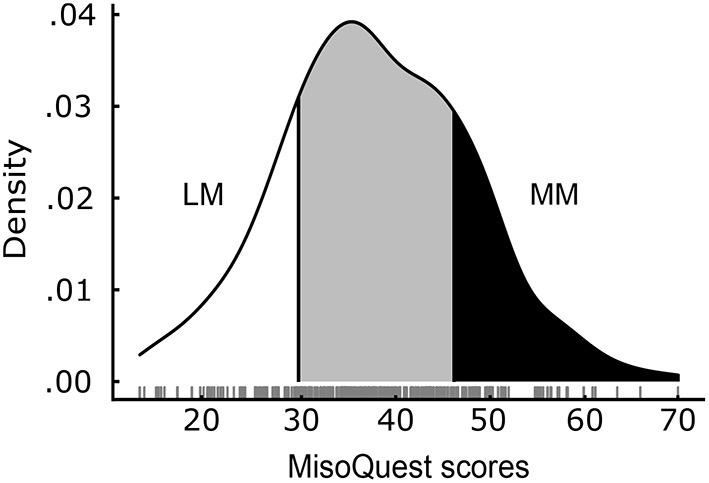
Distribution of MisoQuest scores (*N* = 300). Least-Misophonic (LM) and Most-Misophonic (MM) groups represent the top and bottom 20% of the distribution. Actual scores are plotted below the curve (jittered for better visualization).

Scores on the MisoQuest were normally distributed (minimum: 14, maximum: 69, *M* = 37.9 *SD* = 9.9), as evidenced by a Shapiro–Wilk test supporting the normality of the distribution (*p* = 0.560) and by indices of skewness (0.114) and kurtosis (−0.017) approaching zero. Figure 2 shows the distribution of scores for the entire sample. Mean scores on the MisoQuest did not differ between males and females [*t*_(298)_ = −0.67, *p* = 0.506], and both male and female distributions of MisoQuest scores were also respectively normal according to the Shapiro–Wilk test (female: *p* = 0.653; male: *p* = 0.841). In addition, the MisoQuest scores did not correlate with age (*r* = −0.06, *p* = 0.321), which was also true for males and females separately (female: *p* = 0.151; male: *p* = 0.984). In other words, the data suggest that misophonia symptoms are present in people regardless of sex or age, and is best conceptualized as a continuum in a symmetric and mesokurtic distribution of sound sensitivity. The distributions of MisoQuest scores by sex and by age are presented in Appendix A.

Based on the proposition that certain types of misophonic responses may be more common in women than in men (Kılıç et al., 2021), we compared sexes on their responses to individual items of the MisoQuest. For these additional analyses, given the ordinal nature of the data, we used Mann–Whitney *U*-tests. Results of the tests (using a Bayesian approach) on each item are provided in Appendix B. We found that females scored generally higher on item 14, which assesses impairments in daily functioning, and also scored higher on three items relating to emotional control (item 1, 2, and 5). Of note, the evidence for a sex difference was especially strong for item 5 (“When I hear unpleasant sounds, I start sensing emotions in my body [e.g., I sweat, feel pain, feel pressure, my muscles tense]”), which refers specifically to the physiological component of emotions.

To compare people with and without severe misophonia symptoms on our different measures, we established two groups based on participants' total scores on the MisoQuest. The groups of Most-Misophonic (MM) and Least-Misophonic (LM) included respectively the top and bottom 20% scorers on the MisoQuest, based on a prevalence of 20% for moderate-to-severe misophonia symptoms reported in past literature (Wu et al., 2014; Zhou et al., 2017). The resulting MM group (*N* = 66, 33 females, mean age: 24.0 *SD* = 5.9) included participants with a total score above 45 on the MisoQuest (*M* = 51.3; SD = 5.0), and the LM group (*N* = 68, 32 females, mean age: 25.1 *SD* = 7.6) included participants with total scores below 31 (*M* = 25.1; SD = 4.6).

The groups did not show statistically significant differences in any of the demographic variables, including age [*t*_(132)_=−0.88, *p* = 0.382], sex [χ^2^_(1)_ = 0.12, *p* = 0.733], number of fluent languages [χ^2^_(4)_ = 1.09, *p* = 0.895], continent of residence [χ^2^_(2)_ = 2.69, *p* = 0.261], employment status [χ^2^_(3)_ = 2.69, *p* = 0.261] and student status [χ^2^_(1)_ = 0.21, *p* = 0.645]. The two groups also did not differ in the audio output they used [χ^2^_(3)_ = 4.91, *p* = 0.179], nor the audio quality they reported [χ^2^_(2)_ = 4.18, *p* = 0.124]. In other words, except from MisoQuest scores, the groups did not differ from one another.

### 3.2. Identification Thresholds

**Figure 3:**
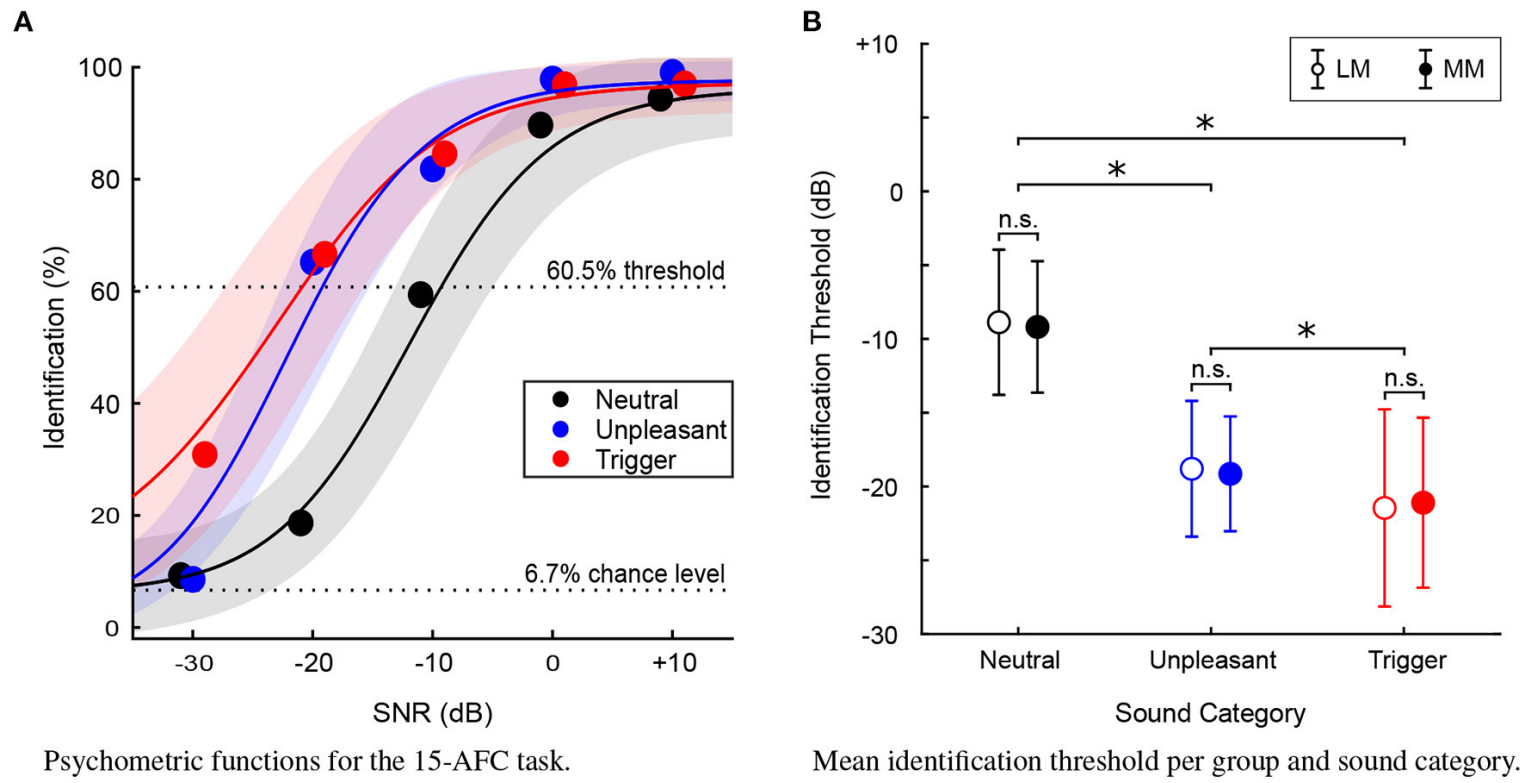
**(A)** Psychometric functions (*N* = 300) for the 15-AFC identification task. Average percent identification plotted at each (SNR) level, for each sound category. Solid lines represent the mean of the fits and shaded areas represent ±1 standard deviation. Dotted lines represent chance level and the performance level chosen to define identification thresholds of the 15-AFC task. **(B)** Mean identification threshold for each sound category, for the Least-Misophonic (LM) and Most-Misophonic (MM) groups. Error bars represent ±1 standard deviation. Asterisk (*) indicates a statistically significant difference (*p* < 0.01), “n.s.” indicates a non-significant difference.

As expected, performance on the identification task averaged over the entire sample (300 participants) increased with SNR, such that when sounds were more easily detectable, performance on the identification task increased to about 100% identification. Percent correctness of sound identification on the 15-AFC-task was used to compute a sigmoidal model of psychometric functions for each category of sound and individual listener (Figure 3A). Although average fit of the models was lower for trigger sounds ($R_{trigger}^{2}$ = 0.912) than for other types of sounds ($R_{unpleasant}^{2}$ = 0.933, $R_{neutral}^{2}$ = 0.937), all goodness of fit indices were above 0.9, and the model fit for each category did not differ between groups.

For each participant, an identification threshold was extracted from the psychometric function of each category of sound. This threshold would represent the SNR level required to attain an arbitrary criterion of 60.5% performance on the identification task for a given sound category. In other words, for each participant, the identification threshold represented the SNR level at which they reliably identified the sounds.

A 2 × 3 mixed ANOVA was conducted to assess differences in identification thresholds between the LM and MM groups for the three sound categories (neutral, unpleasant, trigger). The assumption of sphericity among the three categories of sound was not met. In this analysis, and for all other sets of data that violated this assumption, the degrees of freedom were adjusted with Greenhouse-Geisser [here, χ^2^_(2)_ = 18.8, *p* < 0.001, ϵ = 0.882]. There was a main effect of category [*F*_(1.8, 232.9)_ = 362.8, *p* < 0.001], and *post-hoc* comparisons (with Bonferroni corrections) revealed that thresholds were lower for triggers than unpleasant sounds (by 2.3 dB, *p* < 0.001) which themselves were lower than neutral sounds (by 9.9 dB, *p* < 0.001). However, there was no main effect of Group [*F*_(1, 132)_ = 0.58, *p* = 0.884] nor any interaction of Group and Sound category [*F*_(1.8, 232.9)_ = 0.3, *p* = 0.688]. Figure 3B illustrates the ANOVA results.

The trigger sounds were more salient in general than the other categories of sounds, but this was true of all participants, regardless of whether they were in the LM or MM group. This provides evidence for the first hypothesis, that as SNR increases (and all sounds become easier to detect), there would be a difference in identification of the sounds between sound categories, such that triggers will be identified prior to unpleasant sounds and certainly prior to (at least 10 dB) neutral sounds. It is also evidence against our second hypothesis; individuals most prone to misophonia are not better at detecting trigger sounds than those who are least misophonic.

### 3.3. Subjective Ratings Before and After Identification

**Figure 4:**
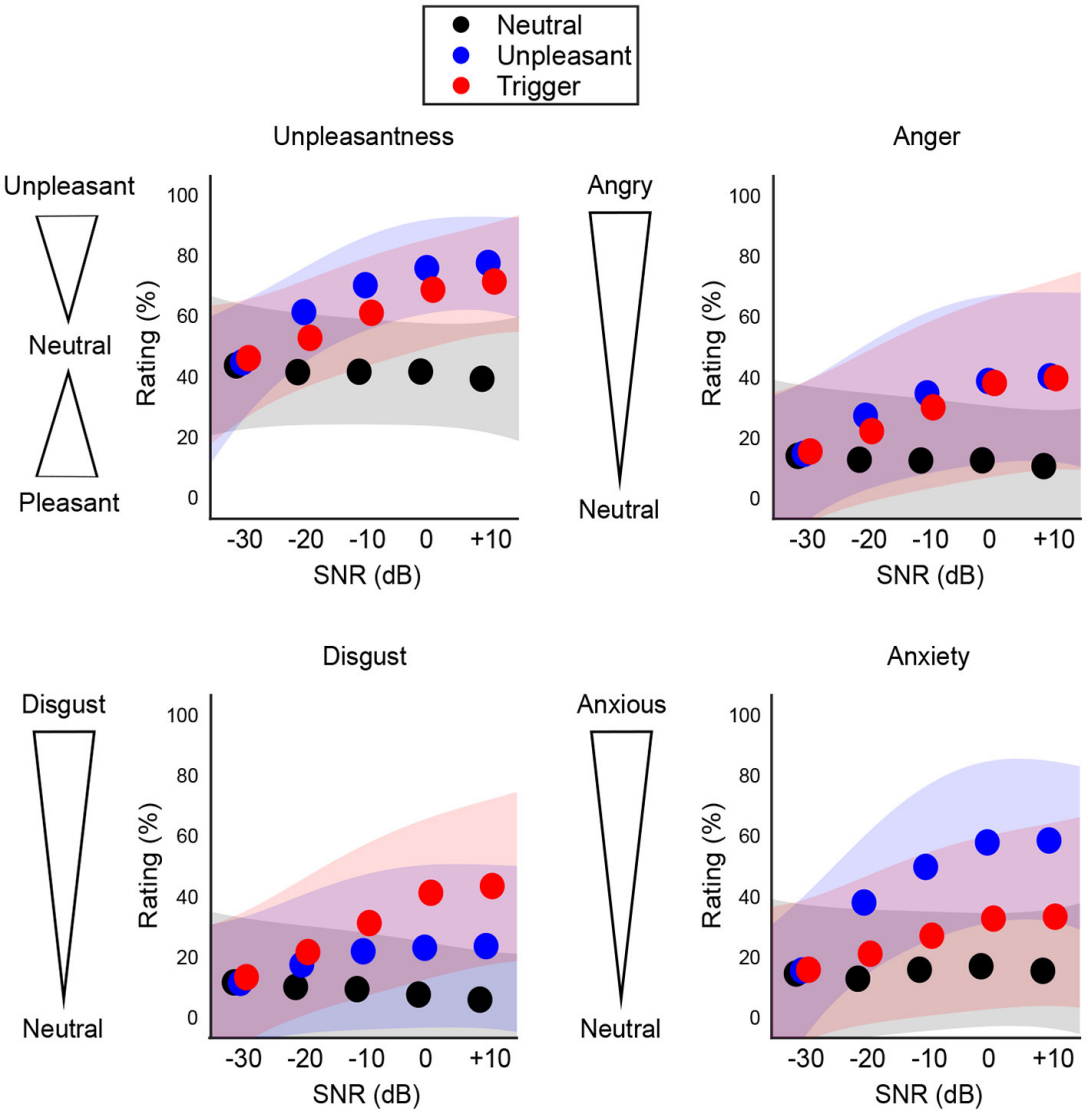
Population results (*N* = 300) for subjective ratings of each sound category. Shaded areas represent ±1 standard deviation from the mean fit. The aversive sounds (blue and red) show a curvilinear trend.

Following a visual assessment of participants' responses for each type of subjective rating as a function of SNR, we observed that the ratings for the aversive sound categories seemed to follow a curvilinear trend. Therefore we used a 2nd-degree polynomial to fit the participant's mean ratings at each level of SNR, for each type of rating and each category of sound (Figure 4).

To assess whether subjective ratings differed in sub-threshold SNRs vs. supra-threshold SNRs, we averaged all points in the subjective fits below and above the threshold to provide only 2 values per category and per participant. For each type of rating, this resulted in a total of six data points per participant: 3 categories of sounds (neutral, unpleasant, trigger) × 2 SNR windows (below recognition threshold, above recognition threshold). Ratings from the self-report scales were flipped, such that high scores indicated a more aversive reaction. Increases in a given rating therefore indicated elevated unpleasantness, anger, disgust, and anxiety.

For each type of rating, we conducted a 2 × 2 × 3 mixed ANOVA, with the within-subject factors being Sound category and SNR window, and the between-subjects factor being Group (LM and MM). For each test, the assumption of sphericity was assessed for Sound category and its interactions; when the assumption of sphericity was not met, degrees of freedom were adjusted with the Greenhouse-Geisser correction. Statistics for main effects and interactions (including effect sizes) are reported in Table 1. Note that, when re-computing these analyses with type of audio output or quality of audio as a between-subjects factor, we found that the main results were not affected. The LM and MM groups did not differ in audio quality or output, the main effect of either of these variables was never significant, and they did not interact with the group variable for any type of rating.

**Table 1:** ANOVA results for emotional ratings before and after recognition threshold.

			**Unpleasantness**	**Anger**	**Disgust**	**Anxiety**
Mauchly's test of sphericity^*a*^						
	Sound category		χ^2^_(2)_ = 12.4, *p* = 0.002, ϵ = 0.917	χ^2^_(2)_ = 2.8, *p* = 0.244	χ^2^_(2)_ = 12.6, *p* = 0.002, ϵ = 0.916	χ^2^_(2)_ = 2.5, *p* = 0.279
	SNR × Sound category		χ^2^_(2)_ = 5.0, *p* = 0.081	χ^2^_(2)_ = 3.2, *p* = 0.200	χ^2^_(2)_ = 3.3, *p* = 0.196	χ^2^_(2)_ = 2.9, *p* = 0.234
Main effects						
	Sound category		*F*_(1.84, 242.16)_ = 272.18	*F*_(2, 264)_ = 135.74	*F*_(1.83, 241.83)_ = 151.49	*F*_(2, 264)_ = 201.91
			*p* < 0.001, η^2^ = 0.110, $\eta_{p}^{2}$ = 0.673	*p* < 0.001, η^2^ = 0.048, $\eta_{p}^{2}$ = 0.507	*p* < 0.001, η^2^ = 0.062, $\eta_{p}^{2}$ = 0.534	*p* < 0.001, η^2^ = 0.076, $\eta_{p}^{2}$ = 0.605
	SNR		*F*_(1, 132)_ = 190.44,	*F*_(1, 132)_ = 143.29	*F*_(1, 132)_ = 113.69	*F*_(1, 132)_ = 193.11
			*p* < 0.001, η^2^ = 0.116, $\eta_{p}^{2}$ = 0.591	*p* < 0.001, η^2^ = 0.068, $\eta_{p}^{2}$ = 0.521	*p* < 0.001, η^2^ = 0.046, $\eta_{p}^{2}$ = 0.463	*p* < 0.001, η^2^ = 0.107, $\eta_{p}^{2}$ = 0.594
	Group		*F*_(1, 132)_ = 0.15	*F*_(1, 132)_ = 16.86	*F*_(1, 132)_ = 16.06	*F*_(1, 132)_ = 19.95
			*p* = 0.702	*p* < 0.001, η^2^ = 0.113, $\eta_{p}^{2}$ = 0.113	*p* < 0.001, η^2^ = 0.073, $\eta_{p}^{2}$ = 0.108	*p* < 0.001, η^2^ = 0.074, $\eta_{p}^{2}$ = 131
2-way interactions						
	SNR × Sound category		*F*_(2, 264)_ = 274.5	*F*_(2, 264)_ = 150.44	*F*_(2, 264)_ = 170.25	*F*_(2, 264)_ = 234.79
			*p* < 0.001, η^2^ = 0.089, $\eta_{p}^{2}$ = 0.675	*p* < 0.001, η^2^ = 0.045, $\eta_{p}^{2}$ = 0.533	*p* < 0.001, η^2^ = 0.057, $\eta_{p}^{2}$ = 0.563	*p* < 0.001, η^2^ = 0.072, $\eta_{p}^{2}$ = 0.640
	SNR × Group		*F*_(1, 132)_ = 6.64	*F*_(1, 132)_ = 17.76	*F*_(1, 132)_ = 8.414	*F*_(1, 132)_ = 17.18
			*p* = 0.011, η^2^ = 0.004, $\eta_{p}^{2}$ = 0.048	*p* < 0.001, η^2^ = 0.008, $\eta_{p}^{2}$ = 0.119	*p* < 0.001, η^2^ = 0.004, $\eta_{p}^{2}$ = 0.060	*p* < 0.001, η^2^ = 0.010, $\eta_{p}^{2}$ = 0.115
	Sound category × Group		*F*_(1.84, 242.16)_ = 4.95	*F*_(2, 264)_ = 11.36	*F*_(1.83, 241.83)_ = 6.41	*F*_(2, 264)_ = 8.93
			*p* = 0.010, η^2^ = 0.002, $\eta_{p}^{2}$ = 0.036	*p* < 0.001, η^2^ = 0.004, $\eta_{p}^{2}$ = 0.079	*p* < 0.003, η^2^ = 0.003, $\eta_{p}^{2}$ = 0.046	*p* < 0.001, η^2^ = 0.003, $\eta_{p}^{2}$ = 0.063
3-way interaction						
	SNR × Sound category × Group		*F*_(2, 264)_ = 8.42	*F*_(2, 264)_ = 12.34	*F*_(2, 264)_ = 7.39	*F*_(2, 264)_ = 8.55
			*p* < 0.001, η^2^ = 0.003, $\eta_{p}^{2}$ = 0.060	*p* < 0.001, η^2^ = 0.004, $\eta_{p}^{2}$ = 0.085	*p* < 0.001, η^2^ = 0.002, $\eta_{p}^{2}$ = 0.053	*p* < 0.001, η^2^ = 0.003, $\eta_{p}^{2}$ = 0.061

*Each column represents a different rating. All ratings showed a similar pattern of results*.

a *Greenhouse-Geisser correction (ϵ) applied when sphericity was violated*.

In addition, we reiterated a similar analysis looking at the linear slope with which subjective ratings degraded as a function of SNR. The results (reported in Appendix C) were largely consistent with those presented here in Section 3.3. This additional analysis reflected the differential trends in how sounds became more aversive as they progressively stood out from the multitalker babble.

#### 3.3.1. Unpleasantness

The first ANOVA revealed a main effect of Sound category and of SNR window, but no main effect of Group. All 2- and 3-way interactions were significant (see Table 1 for statistics). *Post-hoc* comparisons of the 3-way interaction (with Tukey correction for multiple comparisons) revealed that, for each sound category, there was no statistically significant group difference in unpleasantness ratings below threshold (all *p* > 0.999), suggesting that the ratings for the two groups did not significantly differ when they could not detect the sounds.

The average fit of the second-degree polynomial was lower for neutral sounds ($R_{neutral}^{2}$ = 0.656) than for trigger sounds ($R_{trigger}^{2}$ = 0.800), which was itself lower than for unpleasant sounds ($R_{unpleasant}^{2}$ = 0.870). The goodness of fit did not differ between groups.

**Figure 5:**
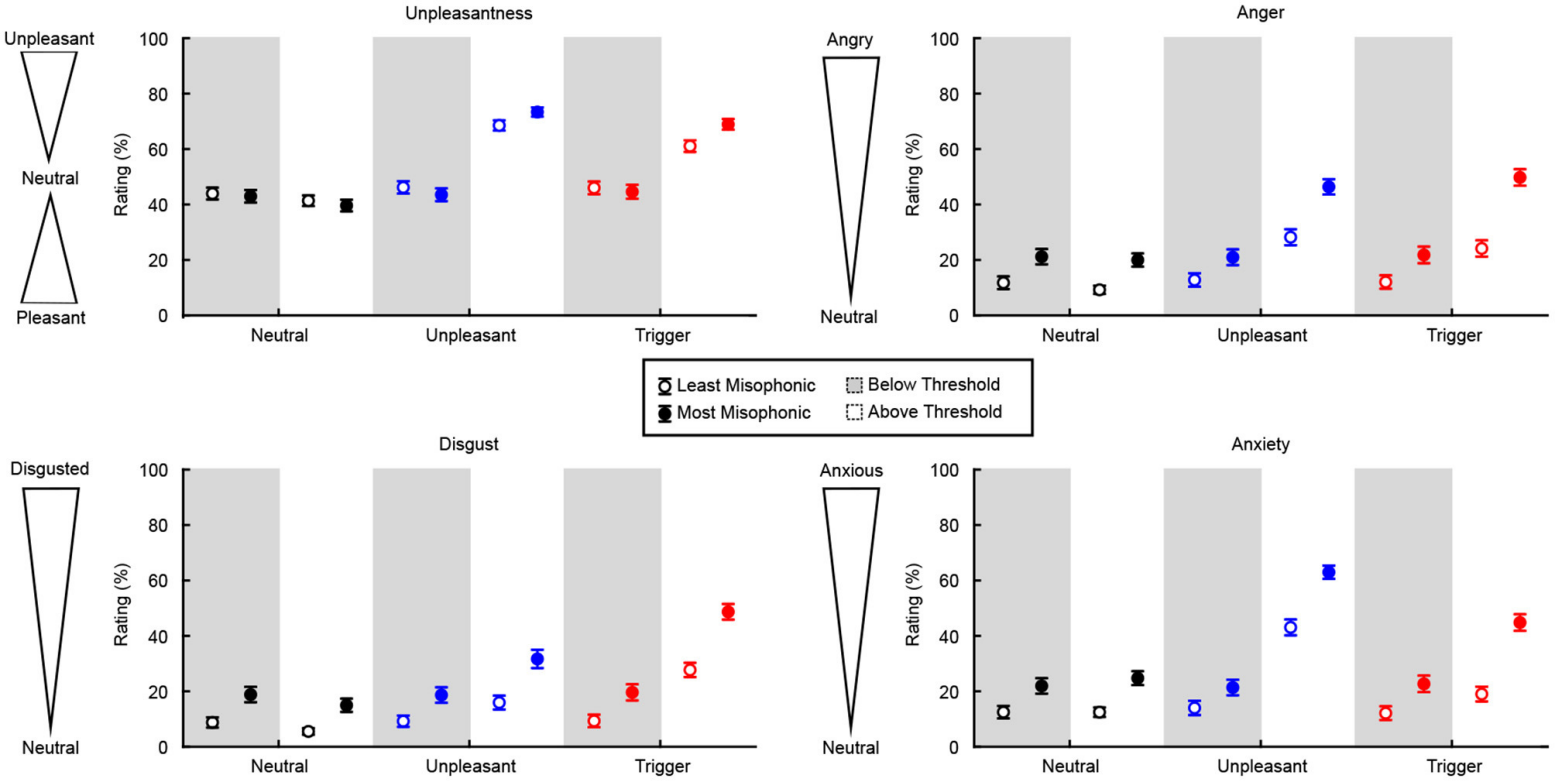
Change in subjective ratings below and above identification thresholds, for each group and sound category. Unpleasantness ratings are from 0 (pleasant) to 100 (unpleasant), with 50 being a neutral rating. For Anger, Disgust, and Anxiety, ratings are from 0 (neutral) to 100. Error bars represent ±1 standard deviation.

To assess the 3-way interaction, we tested specifically the change in rating below and above threshold, across the two groups and the three categories (reducing the design to a 2-way mixed ANOVA). For the LM group, the increase in rating was considerably stronger for unpleasant and trigger sounds relative to neutral sounds (by 24.9 and 17.7 points, *p* < 0.001 in both cases), but the increase was smaller in triggers than in unpleasant sounds (by 7.3 points, *p* = 0.001). For the MM group, the increase in rating was even stronger for unpleasant and trigger sounds than neutral (by 33.2 and 27.7 points, *p* < 0.001 in both cases) and the increase was again smaller for triggers than unpleasant sounds (by 7.3 points, *p* = 0.050). Results of the Unpleasantness ratings below and above the identification threshold, for each sound category and group, are shown in Figure 5. These results illustrate how both groups recognized the unpleasantness of the trigger and unpleasant sounds, but only when the sounds were identified.

Perhaps most importantly, the simple effect of Group on the below/above change in rating was significant for triggers (*p* < 0.001) and unpleasant sounds (*p* = 0.006) but not for neutral sounds (*p* = 0.760). Trigger sounds were the category of sounds where the MM group increased their rating considerably more than the LM group (effect size *d* = 0.64), whereas this was true to a smaller degree for unpleasant sounds (*d* = 0.48), and not true for neutral sounds (i.e., same trend for the two groups). In other words, the increase in unpleasantness ratings for all aversive sounds was more extreme for the MM group than for the LM group, especially for trigger sounds.

#### 3.3.2. Anger

There was a main effect of Sound category, SNR window, and Group, on ratings of Anger. Like for Unpleasantness, all 2- and 3-way interactions were significant. Statistics (including effect sizes) are reported in Table 1. *Post-hoc* comparisons of the 3-way interaction (with Tukey correction for multiple comparisons) revealed that, for each sound category, there was no statistically significant group difference in anger ratings below threshold (all *p* > 0.271), suggesting that the ratings for the two groups did not significantly differ when they could not detect the sounds.

The average fit of the second-degree polynomial was lower for neutral sounds ($R_{neutral}^{2}$ = 0.593) than for unpleasant and trigger sounds ($R_{unpleasant}^{2}$ = 0.768, $R_{trigger}^{2}$ = 0.767), which themselves did not differ from one another. The goodness of fit did not differ between groups.

To assess the 3-way interaction, we again reduced the design to a 2-way mixed ANOVA assessing the change in rating below and above threshold. For the LM group, the elevated anger was considerably stronger for unpleasant and trigger sounds relative to neutral sounds (by 17.97 and 14.65 points, *p* < 0.001 in both cases), but it was not significantly different between triggers and unpleasant sounds. For the MM group, the elevated anger was even stronger for unpleasant and trigger sounds than for neutral sounds (by 26.59 and 29.17 points, *p* < 0.001 in both cases) and again not significantly different between triggers and unpleasant sounds, as illustrated in Figure 5. These results illustrate how both groups felt more anger in response to the unpleasant and trigger sounds when they were identified.

The simple effect of Group on the elevated anger was significant for triggers (*p* < 0.001) and unpleasant sounds (*p* = 0.005), but not for neutral sounds (*p* = 0.760). Trigger sounds were the category of sounds where the MM group experienced elevated anger considerably more than the LM group (*d* = 0.95), which was also true to a smaller degree for unpleasant sounds (*d* = 0.57), but not true for neutral sounds (i.e., same trend around 0% for the two groups). That is, the increase in anger ratings for all aversive sounds was more extreme for the MM group than for the LM group, a pattern which was especially strong for the trigger sounds.

#### 3.3.3. Disgust

There was a main effect of Sound category, SNR window, and Group, on ratings of Disgust. All 2- and 3-way interactions were significant. Statistics (including effect sizes) are reported in Table 1. *Post-hoc* comparisons of the 3-way interaction (with Tukey correction for multiple comparisons) revealed that, for each sound category, there was no statistically significant group difference in disgust ratings below threshold (all *p* > 0.143), suggesting that the ratings for the two groups did not significantly differ when they could not detect the sounds.

The average fit of the second-degree polynomial was lower for neutral sounds ($R_{neutral}^{2}$= 0.581) than for unpleasant sounds ($R_{unpleasant}^{2}$= 0.674), which were themselves lower than for trigger sounds ($R_{trigger}^{2}$= 0.803). The goodness of fit did not differ between groups.

To assess the 3-way interaction, we again reduced the design to a 2-way mixed ANOVA assessing the change in rating below and above threshold. For the LM group, the elevated disgust was considerably stronger for unpleasant and trigger sounds relative to neutral sounds (by 9.96 and 21.65 points, *p* < 0.001 in both cases), and stronger for trigger relative to unpleasant sounds (by 11.69, *p* < 0.001). For the MM group, the elevated disgust was even stronger for unpleasant and trigger sounds than for neutral sounds (by 16.89 and 32.92 points, *p* < 0.001 in both cases), and stronger for triggers than unpleasant sounds (by 16.03 points, *p* < 0.001), as illustrated in Figure 5. These results illustrate how both groups felt more disgust toward the unpleasant and trigger sounds (especially the trigger sounds) when they were identified.

The simple effect of Group on the elevated disgust was significant for triggers (*p* < 0.001), but not for unpleasant (*p* = 0.201) or neutral sounds (*p* = 1.000). Triggers were the category of sounds where the MM group experienced elevated disgust considerably more than the LM group (*d* = 0.60), whereas this was not true for unpleasant sounds and for neutral sounds (i.e., same trend [10–15% increase] for the two groups). In other words, the increase in disgust ratings was more extreme for the MM group than for the LM group, specifically for the trigger sounds.

#### 3.3.4. Anxiety

There was a main effect of Sound category, SNR window, and Group, on ratings of Anxiety. Like for all other types of ratings, all 2- and 3-way interactions were significant. Statistics (including effect sizes) are reported in Table 1. *Post-hoc* comparisons of the 3-way interaction (with Tukey correction for multiple comparisons) revealed that, for each sound category, there was no statistically significant group difference in anxiety ratings below threshold (all *p* > 0.157), suggesting that the ratings for the two groups did not significantly differ when they could not detect the sounds.

The average fit was lower for neutral sounds ($R_{neutral}^{2}$ = 0.606) than for trigger sounds ($R_{trigger}^{2}$ = 0.679), which was itself lower than for unpleasant sounds ($R_{unpleasant}^{2}$ = 0.862). The goodness of fit did not differ between groups.

To assess the 3-way interaction, we again reduced the design to a 2-way mixed ANOVA assessing the change in rating below and above threshold. For the LM group, the elevated anxiety was considerably stronger for unpleasant and trigger sounds relative to neutral sounds (by 29.08 and 6.91 points, *p* < 0.001 and *p* = 0.030), but was weaker for trigger than unpleasant sounds (by 22.17, *p* < 0.001). For the MM group, the elevated anxiety was even stronger for unpleasant and trigger sounds than neutral (by 38.79 and 19.33 points, *p* < 0.001 in both cases), and stronger for unpleasant than trigger sounds (by 19.46, *p* < 0.001), as illustrated in Figure 5. These results illustrate how both groups felt more anxiety toward the unpleasant and trigger sounds (especially the unpleasant sounds) when they were identified.

The simple effect of Group on the elevated anxiety was significant for triggers (*p* < 0.001) and unpleasant sounds (*p* = 0.005), but not neutral sounds (*p* = 1.000). Trigger sounds were the category of sounds where the MM group experienced elevated anxiety considerably more than the LM group (*d* = 0.96), which was also true to a smaller degree for unpleasant sounds (*d* = 0.61), and not true for neutral sounds (i.e., same trend for the two groups). That is to say, the increase in anxiety ratings was more extreme for the MM group than for the LM group for all aversive sounds. Even though the unpleasant sounds generally induced more anxiety once identified, this pattern (the MM group having a stronger increase in anxiety ratings than the LM group) was more extreme of the trigger than unpleasant sounds.

## 4. Discussion

### 4.1. Distribution of Misophonia Symptoms

To characterize the distribution of misophonia symptoms in a general population, we collected responses from an online community sample of 300 participants on the MisoQuest (Siepsiak et al., 2020a). We found that MisoQuest scores were normally distributed (Figure 2), in line with the idea that many people without clinically-significant symptoms still experience negative emotional and physiological reactions to sounds. Some of the sounds that frequently bother people include fingernails scratching on a chalkboard, metal scraping glass, and even some typical misophonic triggers such as chewing or sucking noises (Zald and Pardo, 2002; Kumar et al., 2008). Previous work found that, when using a misophonia-specific scale, a relatively large proportion of the population (68%) reported experiencing such sub-clinical misophonia symptoms (Zhou et al., 2017). In their study, people with sub-clinical symptoms were defined as individuals experiencing misophonia symptoms which did not cause significant distress in daily life. Taken together with the distribution of MisoQuest scores found in this study, it appears that mild misophonia regularly occurs in a large number of people. This observation gives weight to the idea that those who experience daily life impairments as a result of misophonia simply represent the tail end of a normal distribution of misophonia symptoms.

In the development of the MisoQuest, a general cut-off of 61 out of 70 points was proposed to screen for misophonia (Siepsiak et al., 2020a), based on the mean score (minus the standard deviation) of participants self-reporting as having misophonia. Research assessing the psychometric properties of the MisoQuest, found that the questionnaire had good specificity (ability to correctly classify an individual as not having misophonia), but had low sensitivity (ability to correctly classify an individual as having misophonia) (Siepsiak et al., 2020a; Enzler et al., 2021). In other words, using the suggested cutoff point for the MisoQuest introduces a risk of false negatives. In our online community sample, which did not consist of people recruited on the basis of having misophonia or other hearing sensitivities, only 4 out of 300 participants (less than 2%) scored above 61 on the MisoQuest. This result is considerably lower than previous assessments of misophonia's prevalence (i.e., 12–20%, using semi-structured interviews and other misophonia-specific questionnaires), and illustrates the lack of specificity of the MisoQuest as a whole, which has not yet been validated for use in the general population (Siepsiak et al., 2020b). See Supplementary Material for a data-driven grouping approach which attempted to refine this cutoff, an optimization exercise outside the scope of this paper.

Although the 61-point MisoQuest cutoff appears to capture the most severe cases of misophonia, our observations suggest that the distribution of symptom severity in the population lies on a continuum, analogous to some other disorders (e.g., Autism Spectrum Disorder). Previous works proposed that misophonia represents one end of a specific sound sensitivity spectrum, with on the other end Autonomous Sensory Meridian Response (ASMR), a pleasurable tingling sensation in response to trigger sounds (Barratt et al., 2017; McErlean and Banissy, 2018; Rouw and Erfanian, 2018). The MisoQuest was only designed to assess negative emotions, and therefore cannot reflect both ends of that hypothetical ASMR-to-Misophonia continuum. Nonetheless, it may be suitable to measure an individual's severity of symptoms, as similar tools are used for other disorders that vary considerably in their presentation (e.g., the Autism Quotient in the field of autism; Baron-Cohen et al., 2001).

Many different measures have been developed and used in past years—most notably the A-MISO-S (Schröder et al., 2013) and Misophonia Questionnaire (MQ; Wu et al., 2014)—and keep being introduced in the misophonia literature (e.g., more recently the Duke Misophonia Questionnaire by Rosenthal et al., 2021). We hope that our findings about how the MisoQuest behaves in our online community sample can reveal how this measure relates to other scales assessing misophonia. Given recent consensus from clinical experts on a definition of misophonia (Swedo et al., 2022), understanding how these scales behave similarly or differently across multiple populations, and how they correlate with behavioral and physiological responses to trigger sounds, is crucial to the refinement and generalization of misophonia screening tools.

In addition to understanding the prevalence of the disorder (how many people suffer from misophonia), unequal sampling in past research revealed a need for better understanding of the patients' identity (exactly who suffers from misophonia). Unbalanced sex ratios have, so far, prevented researchers from reaching generalizable conclusions on sex differences. In our balanced data set, we found that both male and female distributions of MisoQuest scores were normal, with averages not statistically different from one another. In addition, when looking at the top and bottom 20% of the distribution separately, we found no difference in the number of males and females in each group. This contradicts previous statements on the possible role of sex in misophonia sensitivity (Wu et al., 2014; Zhou et al., 2017).

While misophonia severity may not differ between sexes overall, Kılıç et al. (2021) noted the possibility that certain types of responses may be more common in women than men. This prompted us to assess sex differences for individual items on the MisoQuest. The one item in particular which stood out as interacting with sex referred to the physiological component of emotions. However, this may not be specific to misophonia: men and women tend to differ in self-reported experiences to negative emotional stimuli, with women reporting higher arousal and negative valence (Šolcová and Lačev, 2017). Yet, these self-reports do not correlate with physiological measures of facial electromyography (muscle activity) and skin conductance, which Šolcová and Lačev (2017) proposed to result from stereotypes and emotional beliefs. Future research on misophonia should include physiological metrics to adjudicate on a possible sex-induced difference in physiology when attending to sounds that are known to affect emotions. Further, if this difference does not appear in physiological measures but is present in self reports, future work should attempt to refine questionnaires or possibly weigh items differently based on sex.

In this study, total MisoQuest score did not correlate with age, which contrasts with Kılıç et al. (2021)'s finding that younger individuals were more likely to have misophonia. One explanation for this discrepancy is likely about characteristics of the sample, as younger (less age-balanced) samples do not generally exhibit an effect of age on misophonia (no age effect found in undergraduate samples for Wu et al., 2014; Zhou et al., 2017). Despite our efforts to obtain a sample representative of a general population, by opening the study to all ages, our participants consisted mostly (80%) of adults between 18 and 29 years-old. Note however that there could be individual trajectories of symptoms improving and worsening over time. Early in misophonia research, Edelstein et al. (2013) observed that while 5 of their 11 participants reported symptoms worsening over time, the same number of people reported symptoms staying the same or improving, as they had learned to better cope with them. If there are counteracting trends such that half of individuals with misophonia improve and the other half worsen, this may be seen as a null effect in population results. We therefore do not rule out an effect of age in misophonia, even though our results do not support it at the population level. Future work could clarify how the evolution of symptoms over the lifespan and thus adjudicate on the prevalence of misophonia across different age groups.

Of note, although our results can be considered somewhat more generalizable than past studies done with samples consisting of undergraduate students, our sample may not be representative of all populations. As outlined in the review by Chandler and Shapiro (2016), there are differences between the general population and online convenience samples. Though their review focused on the crowdsourcing platform MTurk, which is suggested to provide data of a lesser quality than Prolific (Eyal et al., 2021), some of the considerations brought up by Chandler and Shapiro (2016) do apply to our sample. For example, in addition to online samples being of younger age than the general population, the review outlines how some groups are often over- and under-represented in such samples. Our sample is somewhat more diverse than in past research, considering that participants came from a variety of countries of origin (though currently residing in English-speaking countries) and had differing employment and student status. However, the present context of an online community sample should be considered when generalizing observations to the population at large, particularly as we did not obtain information regarding ethnicity nor socioeconomic-status. In addition, the screening questions available in the online platform did not allow for the specific exclusion of participants with a diagnosis of anxiety or depression. As we were concerned that the available more general mental health questions would screen out individuals with misophonia, our population of interest, we used a screening question concerning use of medication to treat symptoms of depression, anxiety, or low mood. Because psychiatric symptoms are often co-morbid with misophonia (Rouw and Erfanian, 2018; Erfanian et al., 2019), it is possible that some of the reported misophonia symptoms or high ratings of anger and anxiety in the most-misophonic group could be partially explained by co-morbid affective disorders.

### 4.2. Misophonia, a Sound-Specific or Person-Specific Disorder?

As highlighted in McGeoch and Rouw (2020), the often highly specific nature of trigger sounds (i.e., repetitive, low frequency, etc.) points to the involvement of bottom-up mechanisms, while the complex behavioral and emotional responses suggest involvement of higher-level (top-down) processes. Here, we used a masking paradigm to explore the nature of top-down and bottom-up processing, and how they interact in misophonia.

When assessing identification thresholds for different sound categories, we found that trigger sounds were better identified than unpleasant and neutral sounds. This observation provides evidence for our first hypothesis, about a difference in the acoustic salience of different sound categories, and indicates that trigger sounds are generally easier to detect than other types of sounds. With the small number of stimuli in our study (5 examples of triggers), this finding is difficult to generalize. The sounds chosen for this study aimed at covering the most typical trigger sounds, which are usually orofacial (i.e., produced by the mouth and face) in nature (Jager et al., 2020). There are, however, many different types of trigger sounds and so, future endeavors may continue exploring the idea that common triggers have distinctive acoustic properties that set them apart from other environmental sounds. A limitation of our study (although we found no effect of this in our sample) involved participants potentially having different audio devices perhaps involving sound quality differences. Future assessments of misophonia taking place on online platforms could aim at standardizing the type of listening device, perhaps through the use of screening tools for headphone-users (e.g., Milne et al., 2021); however, for use with rich, naturalistic stimuli such as those used in this study, minor spectral differences caused by output device are less likely to have an effect than overall differences in sound level. A more fruitful approach might be to complement online studies with relatively large sample sizes yet somewhat looser experimental control such as this one with smaller, highly focused and controlled studies in the laboratory environment.

Contrary to what we had hypothesized, the least- and most-misophonic participants did not differ in their ability to detect trigger sounds, suggesting that bottom-up processes (e.g., those engaged in the salience of certain sounds) may in fact be relatively independent of misophonia. Overall, this is evidence against our second hypothesis. Early misophonia research had previously shown some evidence for differences between people with and without misophonia in low level auditory information processing (Schröder et al., 2014) on auditory event-related potentials (ERPs). During an oddball task using pure tones, the authors observed a diminished N1 component to oddball tones in misophonia patients. One of the reasons suggested for this finding was a potential basic impairment in auditory processing at a low level, given that the N1 peak is linked to early attention to auditory stimuli (Näätänen, 1992; Rinne et al., 2006). If individuals who are most and least prone to misophonia do differ in such basic auditory processes, then this is not reflected in our behavioral data, for any type of sound. Our result, paired with the observation that the misophonic reaction is not associated with absolute hearing threshold or hearing impairments in general (Tyler et al., 2014; Jastreboff and Jastreboff, 2015), offers evidence against misophonia being driven by abnormal bottom-up auditory processes. This interpretation is largely consistent with the work of Kumar et al. (2021), who found involvement of the anterior insula in misophonia, known to be essential to top-down control of action mirroring. Yet, caution should be exerted before completely negating the involvement of (abnormal) bottom-up processes (certain acoustic properties of triggers might elicit a form of pain or aversion, see e.g., Arnal et al., 2019). Nevertheless, our conclusions about bottom-up auditory processes emphasize a departure from hyperacusis (i.e., pain in response to environmental sounds, especially loud sounds), even though it tends to be comorbid with misophonia (Jastreboff and Jastreboff, 2014). In hyperacusis, the discomfort is driven by abnormal responses to the sounds' characteristics while the meaning of the sound is irrelevant (Jastreboff and Hazell, 2008); it therefore contrasts with misophonia, in which the sounds' physical characteristics do not appear to be the main component of the response. Of note, the present study did not assess hyperacusis, and as such it is unknown to what extent it might have impacted our results. Still, our observations may indicate that treatment options used in disorders such as hyperacusis would be less effective for misophonia, and that misophonia may respond better to other approaches such as regular counseling (Jastreboff and Jastreboff, 2014).

Certain sounds are more aversive than others; this is true regardless of whether a person has misophonia or not. Often, aversive reactions to sounds depend on their physical properties; for example, generally aversive sounds are loud, rough, and have strong representation of high frequencies (Halpern et al., 1986). However, certain reactions to aversive sounds are based on emotional connections with the sound (Reuter et al., 2014), and thus involve learned associations (top-down processes). In this study, we found that external evaluations of the sounds (unpleasantness) and internal evaluations of emotions (anger, disgust, anxiety) largely paralleled each other, and both appeared only after the sounds were recognized. This parallel suggests that there is a common process to both sound appraisal and personal experience that depends on higher-level cognitive processes. The difference in ratings observed between groups, on trials where the sounds could be identified, thus relates to a higher-level evaluation of the sounds, which is evidence for our third hypothesis. These observations about the involvement of top-down processes are in line with recent findings by Hansen et al. (2021) who showed, using self-report data, that knowledge of the sound identity contributes to the discomfort experienced by people with misophonia. Using a similar design (participants identified sounds and provided aversiveness ratings), they showed that participants who correctly identified oral-nasal sounds rated them as more unpleasant and evoking more discomfort than those who could not identify them.

In our study, on trials where the sounds were identified, the most-misophonic group experienced a stronger increase in aversiveness ratings than the least-misophonic group. This was true to some degree of unpleasant sounds, but it was exacerbated for trigger sounds. For example, the elevated anger and anxiety induced by recognizable trigger sounds was almost 3 times larger for MM than LM individuals. Expressed differently, all participants experienced some discomfort, but participants with higher misophonia symptoms were bothered to a more extreme degree, and with specificity with regard to triggers as opposed to other unpleasant sounds. This finding of exaggerated responses in the MM group when the sounds are identified provides once again evidence for a strong cognitive component in the nature of the misophonic response; there must be something about the meaning of the sound that triggers the response. Differences at higher-level processing between those with and without misophonia are evident from studies using functional brain imaging (Kumar et al., 2017; Schröder et al., 2019). When listening to trigger sounds, participants with misophonia showed abnormal functional connectivity between the anterior insular cortex, critical in perception of interoceptive signals (i.e., signals originating from inside the body) and the default mode network, which includes regions responsible for emotion processing and attending to behaviorally-relevant stimuli. Such differences in brain networks between people with and without misophonia support the idea that memories and contextual associations are strongly tied to the aversive emotions experienced in response to triggers. Together, findings about top-down processing in misophonia call for more behavioral experiments manipulating top-down processes (perhaps manipulating the focus of attention, or instead, the presence of distracting tasks or stimuli) while observing neural correlates to different sound categories.

Here, we provided additional evidence for the idea that cognitive processes, specifically learned associations with identifiable triggers, are involved in misophonia. Treatment options could therefore focus on breaking the associative link with specific triggers. Such treatment options, aimed at treating the cognitive element of the misophonic response, have been anecdotally successful. Although this is often limited to case-studies, cognitive-behavioral therapy (CBT) seems to be effective in reducing misophonia symptoms (Bernstein et al., 2013; McGuire et al., 2015) and managing levels of anger when exposed to triggers (Roushani and Honarmand, 2021). Perhaps more convincingly, Schröder et al. (2017) showed that 48% of patients (*N* = 90) reported a reduction of misophonia symptoms following CBT, whereas the waiting-list control group showed no reduction of misophonia. These results were observed after 3 months of treatment (short-term) and maintained a year later (long-term). The present results, emphasizing a person-centered disorder with a high specificity to certain triggers (not so much other unpleasant sounds) that need to be presented at a sufficiently large SNR to be recognizable, are in full support of such treatment options.

To summarize, in a study involving 300 adults sampled from an online community, two sub-groups of participants were formed on the basis of their self reports in a questionnaire designed for misophonia symptom assessment: a least-misophonic group, largely immune to the impact of sound on their life and wellbeing, and a most-misophonic group that exhibited heightened sensitivity to sound. They all listened to three categories of sounds: neutral sounds, unpleasant sounds (typically aversive), and sounds typically triggering to individuals with misophonia (often orofacially-generated). These sounds, embedded in a multi-talker babble, were presented at different signal-to-noise ratios from very faint in the background (and thus barely identifiable) to perceptually salient (and thus clearly identifiable). Triggers were found to be recognized at a lower SNR than unpleasant sounds and neutral sounds, but this pattern was common in both the least-misophonic and most-misophonic groups. Listeners also rated each sound (identified or not) on four scales: unpleasantness, anger, disgust, and anxiety. As SNR increased, unpleasant and trigger sounds became more aversive (as expected), but this change was more pronounced for triggers than unpleasant sounds, and exacerbated in MM compared to LM individuals. These results demonstrate that the heightened sensitivity of individuals most prone to misophonia does not generalize to sound overall (neutral sounds or sub-threshold unpleasant/trigger sounds). In fact, it does not provide any detection or discrimination advantage, and relates to (conscious) appraisal as well as internal experience of certain triggers, provided that they are sufficiently salient. This pattern of findings strongly supports a role for higher-order processes related to sound identity (and likely its associations with the people generating them, contexts, and so on).

## 5. Conclusion

Misophonia is increasingly recognized as a problem that can significantly affect the wellbeing, education, and careers of sufferers. To devise effective mitigation strategies and effective treatments, we must better understand its prevalence, causes, and physiological basis. This study adds several pieces of information to our knowledge of misophonia. Overall symptom severity was found in a continuum, and was approximately equal in males and females. Although females rated some questionnaire items concerning subjective experiences of physiological responses higher, previous work showed that while males and females might self-report their emotional experiences differently, their physiological responses to negative emotional stimuli do not generally differ (Šolcová and Lačev, 2017). These observations suggests that the biological basis of misophonia is not strongly sex-related, and so eventual treatments might be predicted to work equally well for both males and females. In addition, we demonstrate that while people detect negative and trigger sounds better than neutral sounds in noise, suggesting that those sounds are more salient, people with stronger misophonia symptoms did not show an additional degree of sensitivity for detecting sounds. Conversely, once they were able to identify the aversive sounds, they had a stronger increase of negative emotional reactions to them, particularly for the trigger sounds. Together, these results further emphasize that consciously linking sounds to past experience plays an important role in misophonia.

As described above, the present study has several limitations, one of which being that the questionnaire used (MisoQuest) was validated in a Polish-speaking population (Siepsiak et al., 2020a). While the original authors provided an English translation, and the questionnaire (in English and translated in French) was recently used in a French sample (Enzler et al., 2021), it has not yet been validated in an English-speaking population. To our knowledge, this is the first study that is using the English translation of the questionnaire on English-speakers. In our sample, there was a relatively low proportion of participants who reached the recommended screening score for severe misophonia in our sample, with only 4 participants scoring above 61. This small number is difficult to interpret; because we excluded individuals who were taking psychotropic medications, our distribution may reflect the removal of some more severe cases. This exclusion may reduce the generalizability of our finding to more complex psychiatric patients. However, it did allow us to focus on misophonia symptoms in people whose physiology is not being modulated by pharmaceutics, and to highlight the continuous nature of misophonia severity in a sample more representative of a general population. Given these limitations, we support the proposition by Enzler et al. (2021) that the MisoQuest should be used with other measures of misophonia, to determine potential cut-offs for mild, moderate, and severe symptoms, and to determine the convergent validity of the MisoQuest with other misophonia assessment tools. As regards our experimental design, we chose to use an existing set of stimuli that focuses on orofacial trigger sounds and was used in previous research (Kumar et al., 2017, 2021). Misophonic trigger sounds are not all orofacially generated (the importance of other sources is highlighted in Hansen et al., 2021), although most people with misophonia do have at least one orofacially generated trigger sound (Jager et al., 2020). While a reasonable starting point for fundamental research, an exclusive focus on orofacial sounds across studies could lead to an incomplete mechanistic understanding of misophonia. Therefore, work is needed to characterize the full range of misophonic trigger sounds and produce a wider selection of high-quality stimuli for further study. In addition, although previous research has found similar experiences with misophonia in different cultures (Zhou et al., 2017), the lack of information on ethnicity and socioeconomic-status in our sample should be considered when generalizing our results. Finally, the online study design trades off the precise experimental control over listening contexts and sound quality that are possible in the laboratory with the advantages of being able to recruit a larger sample with an even representation of males and females. While the design appeared to be appropriate for the current questions, which concern perception and recognition of sounds in noise, some research questions such as those requiring fine characterization of individuals' psychiatric profiles and perceptual abilities require an in-person design.

Our main goal was to explore one aspect of misophonia: its relation to identification and memory. Further work is underway to explore physiological markers of these aversive responses, and manipulate listeners' attention to emphasize or deemphasize these sounds' salience. These next studies will be able to inform shorter-term attention-based coping strategies for people living with misophonia. However, attention-based strategies are likely to be effortful and tiring to the user and may represent only a partial solution. More work will be needed to clarify the etiology of misophonia and its evolution across the lifespan, to distinguish preexisting anatomical differences that might predispose people to misophonia from the effects of experience (Kumar et al., 2021), and perhaps to use our knowledge of neuroplasticity within the auditory and motor systems to induce meaningful long-term changes in how people with misphonia process sound (e.g., Herholz and Zatorre, 2012).

## Data Availability Statement

The original contributions presented in the study are publicly available. This data can be found here: https://osf.io/39dzb/.

## Ethics Statement

The studies involving human participants were reviewed and approved by Research Ethics Unit (Office of Research) Concordia University. The patients/participants provided their written informed consent to participate in this study.

## Author Contributions

M-AS, AS, MD, and EC conceived and designed the experiment. M-AS and MD selected and edited stimuli. MD and AS coded the experiment. M-AS, MD, and EC analyzed the data. M-AS drafted the manuscript. All authors reviewed and edited the drafts of the manuscript. All authors contributed to the article and approved the submitted version.

## Funding

This research was supported by the Misophonia Research Fund and REAM foundation, and the Natural Sciences and Engineering Research Council of Canada (NSERC) award to M-AS.

## Conflict of Interest

The authors declare that the research was conducted in the absence of any commercial or financial relationships that could be construed as a potential conflict of interest.

## Publisher's Note

All claims expressed in this article are solely those of the authors and do not necessarily represent those of their affiliated organizations, or those of the publisher, the editors and the reviewers. Any product that may be evaluated in this article, or claim that may be made by its manufacturer, is not guaranteed or endorsed by the publisher.
