# Supplementary material for: Specificity of Affective Responses in Misophonia Depends on Trigger Identification
Source: Front Neurosci. 2022 May 26;16:879583. doi: 10.3389/fnins.2022.879583 (PMC9179422; doi:10.3389/fnins.2022.879583)
Supplement: Supplementary file 1 [file Data_Sheet_1.pdf]

Supplementary material for 'Specificity of Affective Responses in Misophonia Depends on Trigger Identification'

Supplementary Material: Data-driven Approach

Given that the MisoQuest has been developed as a tool to screen for misophonia yet has not been validated in a general population (only in misophonic samples), we employed a data-driven approach to determine whether individual items on the MisoQuest would have yielded different groups of least- and most-misophonic.

With the main feature of misophonia being negative reactions to specific trigger sounds, we reasoned that the most misophonic individuals would show a stronger post-identification increase in negative emotions for trigger sounds than for the generally aversive sounds. Therefore, we computed difference scores (of post-identification change in rating) to be used as a measure of difference in misophonic reaction between unpleasant and trigger sounds. Positive values for difference scores indicated an elevated aversiveness once triggers became identifiable versus once unpleasant sounds became identifiable. For each of the distributions, following a similar approach as for MisoQuest grouping, we then formed groups based on the bottom and top 20% of each distribution. Because there are 4 types of ratings, this yielded 4 grouping approaches. See Figure S1 for the distributions of difference scores.

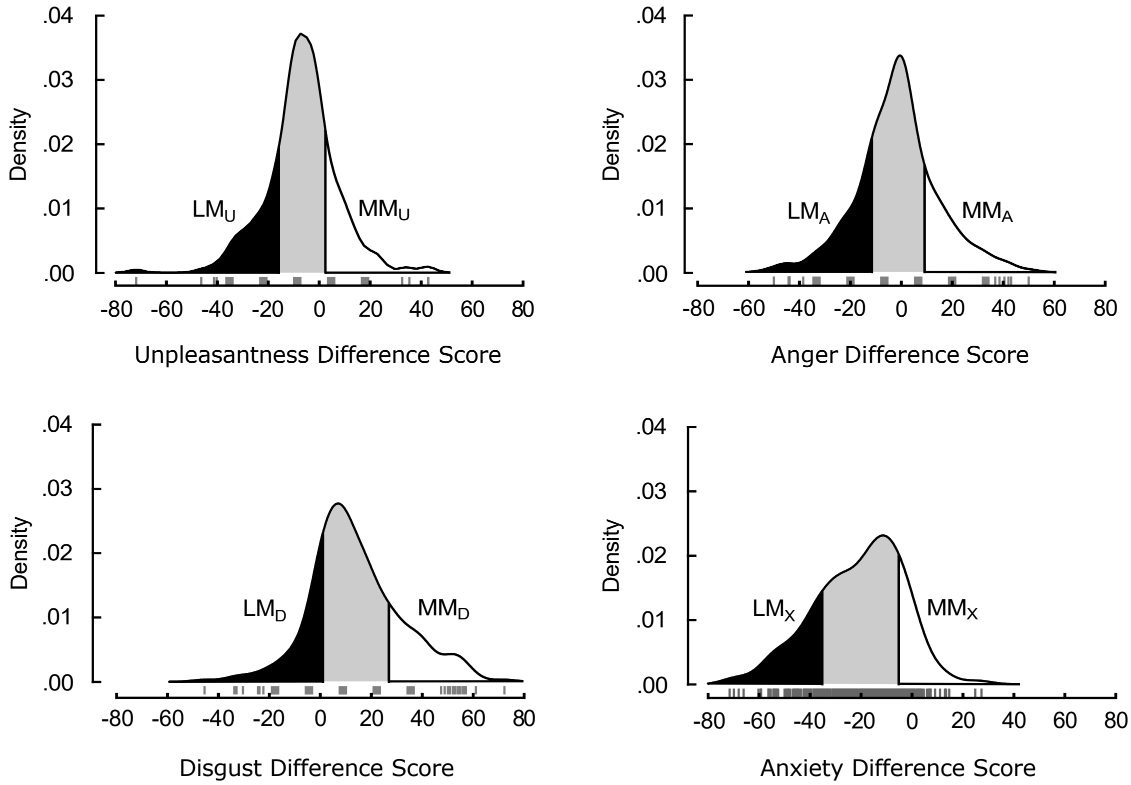

Figure 1: Distribution of difference scores between the increase in rating from below to above recognition threshold for trigger vs unpleasant sounds (N=300). A negative value on the X-axis indicates that the increase in rating from below to above threshold is stronger for unpleasant sounds than for triggers, while a positive value indicates that the increase in rating was stronger for triggers than unpleasant sounds. Least-Misophonic (LM) and Most-Misophonic (MM) groups, formed on the basis of negativity ratings, represent the top and bottom 20% of each distribution. Actual difference scores are plotted below the curves.

Using the new grouping approaches, we examined how the groups differed on individual MisoQuest items. This was done to determine if scoring low or high on specific items could explain differences in behaviour. A Bayesian approach was used used in combination with the traditional NHST approach, to assess evidence for the hypothesis of a group difference on each item. Given the ordinal nature of the data, we used Mann-Whitney U tests. Tables S1-S4 report results of the tests for individual items (including the Bayesian approach).

We found that the groups formed from the Unpleasantness difference scores (LM_U and MM_U) did not differ on any item, as shown in Table 1. The groups based on Anger difference scores (LM_A and MM_A) differed on items 6, 7, 8, and 9, as shown in Table 2. Groups based on Disgust difference scores (LM_D and MM_D) differed on item 13, however significance testing contradicted the Bayesian approach, which offered evidence for the null hypothesis. Finally, groups based on Anxiety difference scores (LM_X and MM_X) differed on item 10.

Based on these observations, we propose that giving more weight to specific MisoQuest items might allow a better reflection of MisoQuest symptoms, and perhaps improve the sensitivity of the MisoQuest by increasing the number of misophonics who meet the proposed cut-off.

Table 1: Mann-Whitney U tests for an effect of group on individual items of the MisoQuest. The Least-Misophonic (LM_U) and Most-Misophonic (MM_U) groups were formed according to the **Unpleasantness-driven grouping**. Both the results of NHST and Bayesian tests are reported.

	Significance Testing			Bayesian approach
	W	*p*	Rank-Biserial Correlation	BF10
MQ1	2031.0	.184	.000	0.325
MQ2	1978.5	.325	.099	0.269
MQ3	1669.5	.459	-.073	0.232
MQ4	1651.0	.412	-.083	0.222
MQ5	1875.0	.681	.042	0.213
MQ6	2070.0	.132	.150	0.352
MQ7	2033.5	.201	.130	0.334
MQ8	2141.5	.062	.190	0.579
MQ9	2062.0	.150	.146	0.461
MQ10	2141.0	.057	.189	0.665
MQ11	1719.5	.663	-.045	0.214
MQ12	1803.0	.989	.002	0.206
MQ13	1991.5	.272	.106	0.264
MQ14	1726.0	.681	-.041	0.206

Table 2: Mann-Whitney U tests for an effect of group on individual items of the MisoQuest. The Least-Misophonic (LM_A) and Most-Misophonic (MM_A) groups were formed according to the **Anger-driven grouping**. Both the results of NHST and Bayesian tests are reported.

	Significance Testing			Bayesian approach
	W	*p*	Rank-Biserial Correlation	BF10
MQ1	1932.0	.451	.073	0.295
MQ2	2165.0	.040	.203	0.957
MQ3	1809.5	.959	.005	0.214
MQ4	1991.5	.291	.106	0.957
MQ5	2072.0	.133	.151	0.479
MQ6	**2230.0**	**.016**	**.239**	**1.163**
MQ7	**2222.0**	**.021**	**.234**	**1.056**
MQ8	**2244.0**	**.014**	**.247**	**2.378**
MQ9	**2219.0**	**.022**	**.233**	**1.909**
MQ10	2037.0	.187	.132	0.410
MQ11	1724.0	.679	-.042	0.210
MQ12	1745.0	.765	-.031	0.208
MQ13	1743.0	.743	-.032	0.224
MQ14	1884.5	.637	.047	0.241

Table 3: Mann-Whitney U tests for an effect of group on individual items of the MisoQuest. The Least-Misophonic (LM_D) and Most-Misophonic (MM_D) groups were formed according to the **Disgust-driven grouping**. Both the results of NHST and Bayesian tests are reported.

	Significance Testing			Bayesian approach
	W	*p*	Rank-Biserial Correlation	BF10
MQ1	2067.5	.129	.149	0.625
MQ2	2109.0	.089	.172	0.585
MQ3	1766.5	.853	-.019	0.213
MQ4	1765.5	.852	-.019	0.214
MQ5	1933.5	.465	.074	0.235
MQ6	1914.0	.530	.063	0.216
MQ7	1811.0	.954	.006	0.203
MQ8	1966.0	.365	.092	0.257
MQ9	1979.0	.329	.099	0.299
MQ10	1861.0	.733	.034	0.222
MQ11	1953.5	.402	.085	0.281
MQ12	1907.5	.562	.060	0.227
MQ13	**1438.5**	**.038**	**-.201**	**0.844**
MQ14	1945.5	.418	.081	0.255

Table 4: Mann-Whitney U tests for an effect of group on individual items of the MisoQuest. The Least-Misophonic (LM_X) and Most-Misophonic (MM_X) groups were formed according to the **Anxiety-driven grouping**. Both the results of NHST and Bayesian tests are reported.

	Significance Testing			Bayesian approach
	W	*p*	Rank-Biserial Correlation	BF10
MQ01	1897.0	.584	.000	0.215
MQ02	1838.5	.833	.021	0.199
MQ03	1841.5	.816	.023	0.200
MQ04	1650.0	.413	-.083	0.230
MQ05	1588.0	.244	-.118	0.336
MQ06	2036.0	.187	.131	0.343
MQ07	1741.0	.749	-.033	0.222
MQ08	1993.0	.296	.107	0.239
MQ09	1957.5	.391	.087	0.263
MQ10	**2236.5**	**.016**	**.242**	**1.530**
MQ11	1714.5	.644	-.047	0.231
MQ12	1706.5	.611	-.052	0.219
MQ13	1977.0	.301	.098	0.315
MQ14	1786.5	.943	-.007	0.206
