# Supplementary material for: Specificity of Affective Responses in Misophonia Depends on Trigger Identification
Source: Front Neurosci. 2022 May 26;16:879583. doi: 10.3389/fnins.2022.879583 (PMC9179422; doi:10.3389/fnins.2022.879583)
Supplement: Supplementary file 2 [file Data_Sheet_2.pdf]

Appendices

1 Figures for MisoQuest scores by Sex and Age

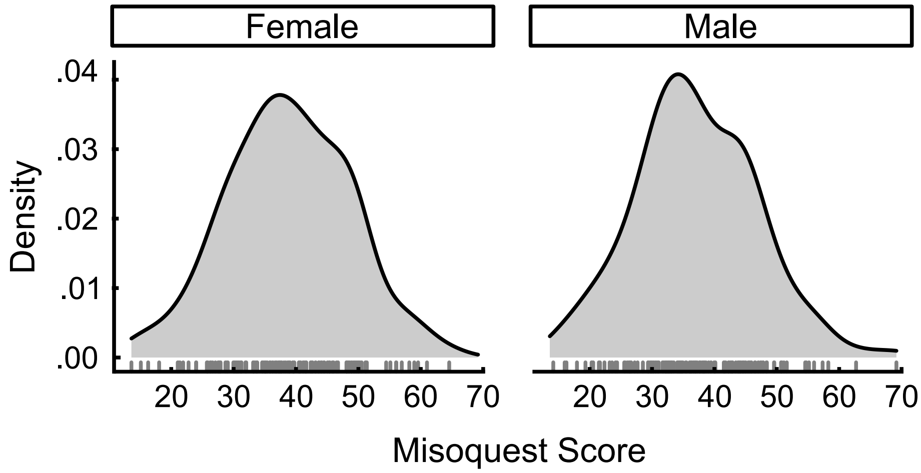

Figure 1: Distribution of MisoQuest scores by sex. Actual scores are plotted below the curve (jittered for better visualization). Both male and female distributions are normal according to the Shapiro-Wilk test (female: *p*=.653; male: *p*=.841).

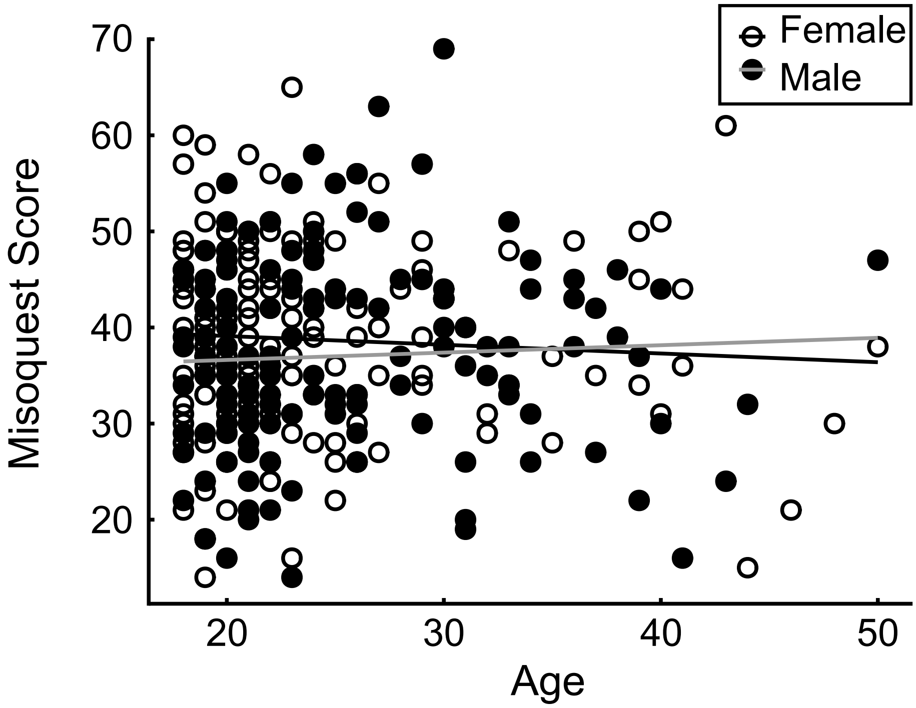

Figure 2: Distribution of MisoQuest scores by age, for each sex. Female: $R^2(149)= .004$, $p= .441$; Male : $R^2(147)<.01$, $p= .534$. All : $R^2(298)= <.01$, $p= .779$. There is no correlation between age and MisoQuest scores in this dataset.

2 MisoQuest Items by Sex

A Bayesian approach was used used here in combination with the traditional NHST approach, to assess evidence for the hypothesis of a group difference on each item. Given the ordinal nature of the data, we used Mann-Whitney U tests.

Table 1: Mann-Whitney U tests for an effect of sex on individual items of the MisoQuest. Both the results of NHST and Bayesian (evidence for specific hypotheses) tests are reported.

	Females		Males			Significance Testing		Bayesian
	M	SD	*M*	*SD*	*W* (298)	*p*	rb[A]	BF10[B]
MQ01	3.40	1.05	3.10	1.08	**13002.0**	**.012**	**.156**	**1.26**
MQ02	3.49	1.08	3.18	1.17	**12878.0**	**.022**	**.145**	**1.08**
MQ03	2.54	1.02	2.44	0.98	1783.0	.449	.047	0.18
MQ04	3.13	1.11	2.95	1.18	12159.0	.205	.081	0.28
MQ05	3.34	1.07	2.88	1.08	**13916.5**	**<.001**	**.237**	**22.61**
MQ06	2.60	1.01	2.57	1.09	11505.0	.719	.023	0.14
MQ07	2.56	1.23	2.48	1.19	11589.5	.637	.030	0.15
MQ08	2.66	1.12	2.77	1.08	10585.5	.359	-.059	0.16
MQ09	2.66	1.26	2.49	1.09	11947.5	.335	.062	0.19
MQ10	1.96	0.94	2.08	1.07	10729.0	.450	-.047	0.18
MQ11	2.98	1.21	3.00	1.16	11185.0	.929	.000	0.13
MQ12	2.82	1.21	2.73	1.21	11722.5	.513	.042	0.15
MQ13	1.62	0.82	1.72	0.81	10355.0	.189	-.080	0.21
MQ14	2.99	1.13	2.63	1.11	**13231.5**	**.006**	**.176**	**2.71**

[A]Effect size given with a rank-biserial correlation coefficient

[B]Evidence for the hypothesis that scores differ between males and females.

3 Linear Slopes of the subjective ratings

Here, we examine the linear trend of the second-degree polynomial with which the subjective ratings were fitted. This linear slope serves as an indicator of how fast subjective ratings change as a function of SNR.

For each type of rating (Unpleasantness, Anger, Disgust, Anxiety), we conducted a 2x3 Mixed ANOVA on the slopes, with the within-subject factors being Sound Category (neutral, unpleasant, trigger) and the between-subjects factor being the Group (LM and MM). For each test, the assumption of sphericity was assessed for sound category and its interactions; when the assumption of sphericity was violated, degrees of freedom were adjusted with the Greenhouse-Geisser correction.

3.0.1 Unpleasantness

The ANOVA revealed that both main effects and the interaction were statistically significant. Statistics (including effect sizes) are reported in Table 2.

Post-hoc tests for the 2-way interaction revealed that for the LM group, the slopes were considerably steeper for unpleasant and trigger sounds relative to neutral sounds [by 5.7 and 5.3% per 10dB, p<.001 in both cases], but not different between unpleasant and trigger sounds [p=1.000]. For the MM group, the increase in rating was even stronger for unpleasant and trigger sounds than neutral [by 8.1 and 7.7% per 10dB, p<.001 in both cases], but again not different between unpleasant and trigger sounds [p=1.000]. Notably, there was no statistically significant difference between the pleasantness slopes of the MM and LM groups for neutral, unpleasant, and trigger sounds, showing that both groups had similar pleasantness slopes in general.

3.0.2 Anger

The ANOVA revealed that both main effects and the interaction were statistically significant. Statistics (including effect sizes) are reported in Table 2.

Post-hoc tests for the 2-way interaction revealed that for the LM group, the slopes were considerably steeper for unpleasant and trigger sounds relative to neutral sounds [by 5.2 and 4.6% per 10dB, p<.001 in both cases], but not different between unpleasant and trigger sounds [p=1.000]. For the MM group, the increase in rating was even stronger for unpleasant and trigger sounds than neutral [by 7.9 and 9.0% per 10dB, p<.001 in both cases], but again not different between unpleasant and trigger sounds [p=1.000]. Notably, the MM group had significantly steeper slopes than the LM group for both unpleasant and trigger sounds [by 3.0 and 4.8% per 10dB, p=.005 and p<.001 respectively], but did not differ in terms of slopes for the neutral sounds.

3.0.3 Disgust

The ANOVA revealed that both main effects and the interaction were statistically significant. Statistics (including effect sizes) are reported in Table 2.

Post-hoc tests for the 2-way interaction revealed that for the LM group, the slopes significantly different for all categories of sounds, such that the slopes for trigger sounds were steeper than for unpleasant sounds [by 3.6% per 10dB, p<.001] which were themselves steeper than for neutral sounds [by 2.9% per 10dB, p<.001]. This was also true for the MM group, for which the slopes for trigger sounds were steeper than for unpleasant sounds by 5.7% per 10dB [p<.001], and the slopes for the unpleasant sounds were themselves steeper than for neutral sounds by 4.8% per 10dB [p<.001]. Notably, the MM group had significantly steeper slopes than the LM group for the trigger sounds only [by 3.5% per 10dB, p<.001], but was not statistically different in terms of slopes for the neutral and unpleasant sounds.

3.0.4 Anxiety

The ANOVA revealed that both main effects and the interaction were statistically significant. Statistics (including effect sizes) are reported in Table 2.

Post-hoc tests for the 2-way interaction revealed that for the LM group, the slopes significantly different for all categories of sounds, such that the slopes for unpleasant sounds were steeper than for trigger sounds [by 6.2% per 10dB, p<.001] which were themselves steeper than for neutral sounds [by 2.0% per 10dB, p=.045]. This was also true for the MM group, for which the slopes for unpleasant sounds were steeper than for trigger sounds by 4.0% per 10dB [p<.001] and the slopes for the trigger sounds were were themselves steeper than for neutral sounds by 6.2% per 10dB [p<.001]. Notably, the MM group had significantly steeper anxiety slopes than the LM group for both unpleasant and trigger

sounds [by 2.9 and 5.1% per 10dB, p=.016 and p<.001 respectively], but did not differ in terms of slopes for the neutral sounds.

Table 2: ANOVA results for linear slopes. Each column represents a different rating.

	Unpleasantness	Anger	Disgust	Anxiety
Mauchly's test of sphericity[1]				
Sound Category	$\chi^2(2) = 4.76$, $p = .093$	$\chi^2(2) = 7.20$, $p = .027$, ε= .949	$\chi^2(2) = 0.41$, $p = .815$	$\chi^2(2) = 1.63$, $p = .444$
Main effects				
Sound Category	$F(2,264) = 132.47$	$F(1.89,250.60) = 127.841$	$F(2,264) = 194.59$	$F(2,264) = 182.72$
	$p < .001$, $\eta^2 = .283$, $\eta_p^2 = .501$	$p < .001$, $\eta^2 = .276$, $\eta_p^2 = .492$	$p < .001$, $\eta^2 = .400$, $\eta_p^2 = .596$	$p < .001$, $\eta^2 = .336$, $\eta_p^2 = .581$
Group	$F(1,132) = 4.31$	$F(1,132) = 18.94$	$F(1,132) = 12.37$	$F(1,132) = 19.20$
	$p = .040$, $\eta^2 = .013$, $\eta_p^2 = .032$	$p < .001$, $\eta^2 = .052$, $\eta_p^2 = .125$	$p < .001$, $\eta^2 = .026$ $\eta_p^2 = .086$	$p < .001$, $\eta^2 = .051$, $\eta_p^2 = .127$
2-way interaction				
Sound Category x Group	$F(2,264) = 4.26$	$F(1.89,250.60) = 10.58$	$F(2,264) = 10.78$	$F(2,264) = 9.38$
	$p = .015$, $\eta^2 = .009$, $\eta_p^2 = .031$	$p < .001$, $\eta^2 = .023$, $\eta_p^2 = .074$	$p < .001$, $\eta^2 = .022$, $\eta_p^2 = .075$	$p < .001$, $\eta^2 = .017$, $\eta_p^2 = .066$

[1]Greenhouse-Geisser correction (ε) applied when sphericity was violated.
